# Association of the systemic inflammation and anthropometric measurements with cancer risk: a prospective study in MJ cohort

**DOI:** 10.3389/fonc.2024.1400893

**Published:** 2024-09-09

**Authors:** Zilong Bian, Luopiao Xu, Yuting Wang, Min-Kuang Tsai, David Ta-Wei Chu, Huakang Tu, Chi-Pang Wen, Xifeng Wu

**Affiliations:** ^1^ Department of Big Data in Health Science School of Public Health, and Center of Clinical Big Data and Analytics of The Second Affiliated Hospital, Zhejiang University School of Medicine, Hangzhou, China; ^2^ Institute of Population Health Sciences, National Health Research Institutes, Zhunan, Taiwan; ^3^ MJ Health Management Center, Taipei, Taiwan; ^4^ National Institute for Data Science in Health and Medicine, Zhejiang University, Hangzhou, China; ^5^ School of Medicine and Health Science, George Washington University, Washington DC, United States

**Keywords:** inflammation, cancer incidence, obesity, cohort, anthropometric

## Abstract

**Objective:**

To investigate the specific role of inflammation in the connection between obesity and the overall incidence of cancer.

**Methods:**

A total of 356,554 participants in MJ cohort study were included. Systemic inflammation markers from blood samples and anthropometric measurements were determined using professional instruments. The Cox model was adopted to evaluate the association.

**Results:**

Over a median follow-up of 8.2 years, 9,048 cancer cases were identified. For individual systemic inflammation biomarkers, the overall cancer risk significantly escalated as blood C-reactive protein (CRP) (hazard ratio (HR)=1.036 (1.017-1.054)) and globulin (GLO) (HR=1.128 (1.105-1.152)) levels increased, and as hemoglobin (HEMO) (HR=0.863 (0.842-0.884)), albumin (ALB) (HR=0.846 (0.829-0.863)) and platelets (PLA) (HR=0.842 (0.827-0.858)) levels decreased. For composite indicators, most of them existed a significant relationship to the overall cancer risk. Most indicators were correlated with the overall cancer and obesity-related cancer risk, but there was a reduction of association with non-obesity related cancer risk. Most of indicators mediated the association between anthropometric measurements and overall cancer risk.

**Conclusions:**

Systemic inflammatory state was significantly associated with increased risks of cancer risk. Inflammation biomarkers were found to partly mediate the association between obesity and cancer risk.

## Introduction

“Tumor-promoting inflammation” plays a fundamental role in activating the hallmark capabilities required for tumor development and progression (1). Inflammation has a great impact on the composition of the tumor microenvironment, primarily by supplying various type of tumor promoting elements such as growth factors, proangiogenic factors, anti-apoptotic and invasion-promoting factors, and chemokines, thus resulting in tumor development (2).

Several systemic inflammation markers have been validated as predictors of cancer incidence. For instance, there have been identified associations between C-reactive protein (CRP) (3–5) and fibrinogen levels (3, 6) and cancer incidence. Few studies focused on composite indicators. In the UK biobank, Nøst et al. uncovered the connections between composite indicators, such as the systemic immune-inflammation index (SII), with the risk of developing malignant neoplastic diseases across 17 different sites (7). A higher SII was also found to be a powerful marker of developing solid cancers in one Rotterdam cohort (8). The significance of this dynamic relationship is particularly notable due to the pivotal role inflammation plays as a reversible mechanism through which obesity influences the risk and advancement of cancer (9).

Obesity is indicative of a persistent subclinical inflammatory condition, and the inflammation of adipose tissue emerges as a pivotal process in the development of obesity-related cancers (10). When adipose tissue exceeds its blood supply, it can enter a state of hypoxia, resulting in adipocyte stress and subsequent cellular demise (11). The strong relationships between obesity and a variety of tumors were mostly attributed the negative impact of inflamed adipose tissue on the tumor microenvironment (12). The association between body mass index (BMI), CRP, and cancer risk has been explored through various Mendelian randomization studies (13, 14). However, the specific role of inflammation in the connection between obesity and the overall incidence of cancer remains unclear.

Thus, in a comprehensive population-based prospective study known as the MJ Cohort, we examined the impact of systemic inflammation and obesity on obesity-related or non-obesity related cancer risk.

## Methods

### Study population

The present study utilized individual data clinical information from the prospective Taiwan MJ cohort study (15–17). The MJ Cohort have been extensively described in previous publications (15–17). In brief, the MJ Health Database was a long-term tracking database established on the basis of a healthy population, including socio-economic, behavioral, and health check biochemical data. From 1996 to the end of 2008, the MJ Health Data Database has accumulated more than 1.5 million health checkups and questionnaire records, with a total of approximately 500,000 participants. Approval for this study was obtained from the institutional review boards of two esteemed organizations in Taiwan, namely the MJ Health Management Institution and the National Health Research Institutes. All participants provided informed consent at baseline assessment.

### Inflammation indicators

CRP, total protein (TP), albumin (ALB), globulin (GLO), platelets (PLA), hemoglobin (HEMO), leukocyte differential counts (including neutrophil, lymphocyte, monocyte) and white blood cells (WBC) counts were considered as biomarkers of systemic inflammation response. WBC counts and leukocyte differential counts were measured using ABBOTT Cell- Dyn 3000 with 4 angle diffraction analysis. CRP, TP, and ALB were analyzed by HITACHI 7150 or TOSHIBA C8000.

We calculated the following related evaluation indicators based on established methods in the literature: Albumin/Globulin (AGR) (18), aggregate index of systemic inflammation (AISI) (19), C-reactive Protein/Albumin (CAR) (20), derived NLR (dNLR) (21), monocyte-lymphocyte ratio (MLR) (22), neutrophil to lymphocyte × platelet ratio (NLPR) (23), neutrophil-to-lymphocyte ratio (NLR) (24), platelet-to-lymphocyte ratio (PLR) (25), Prognostic Nutritional Index (PNI) (26), SII (27) and systemic inflammation response index (SIRI) (28). The concrete calculation formula of the above indicators was shown in **Supplementary Table S1**.

### Anthropometric measurements

Anthropometric measurements conducted in this study encompassed body weight, body height, chest circumference, waist circumference (WC) and hip circumference (HC). Subsequently, additional indexes, including the waist-to-hip ratio (WHR), waist-to-height ratio (WHtR), body mass index (BMI), and A Body Shape Index (ABSI), were computed as part of the analysis. These indexes provide valuable insights into various aspects of body composition and shape, allowing for a comprehensive assessment of participants’ physical characteristics. WHR was computed by dividing WC (in cm) by HC. BMI (kg/m^2^) was defined as the division of body weight (kg) by squared height (m^2^)) (29). ABSI was estimated as WC/(BMI^2/3^×height^1/2^) (30).

The following measurement standards were applied to ensure the accuracy of relevant anthropometric indicators. The Nakamura KN-5000A auto-anthropometer, manufactured by Nakamura in Tokyo, Japan, was utilized to obtain accurate measurements of body weight and height. Body weight and height measurements were taken with precision to the nearest 0.1 kg and 0.1 cm. Barefoot and wearing light indoor clothing were required to ensure accurate results. Waist circumference, measured with precision down to the millimeter, was assessed at the precise midpoint between the lower margin of the rib cage and the crest of the ilium. Similarly, with meticulous precision down to the millimeter, hip circumference was assessed by measuring around the pelvis at the precise location where the buttocks protrude the most (15).

For the assessment of body fat percentage (BF%), we employed foot-to-foot bioelectrical impedance analysis using the TANITA^®^ TBF machines. The measurements were conducted in an upright position. Lean body mass (LBM) (31) was calculated as total body weight minus fat mass.

### Assessment of covariates

Sociodemographic, behavioral risk factors and other confounding factors that have the potential to confound the association between inflammatory markers and cancer incidence were considered as covariates, which included age at recruitment, sex, education level, marital status, smoking, alcohol consumption, physical activity, diet, and family history of cancer. The participants’ education level was categorized as follows: college or above, high school or equivalent, and less than high school. Marital status was categorized into married and unmarried. Smoking and drinking status were classified as never, former, and current. Similar to our previous studies (15–17), the volume of leisure time physical activity was divided into three groups: inactive (less than 3.75 metabolic equivalent (MET) hours/week), low active (ranging from 3.75 to 7.49 MET hours/week), and fully active (more than or equal than 7.50 MET hours/week). Based on dietaries that were linked to inflammation (32), a healthy diet was defined based on the following criteria: consumption of a minimum of 4 out of the following 7 food groups: fruits (≥ 3 servings per day), vegetables (≥ 3 servings per day), fish (≥ 2 times per week), processed meats (≤ 1 time per week), unprocessed meats (≤ 2 times per week), whole grains (≥ 4 servings per day), refined grains (≤ 1 serving per day), and sugar (≤ 1 time per week). We coded missing data as a missing indicator category for categorical variables (33), and used sex-specific medians to impute the missing value for continuous variables. All covariates had <10% missing data.

### Follow-up for cancer incidence

Previous publications have provided comprehensive descriptions of the follow-up procedures (15–17). In summary, comprehensive data on cancer incidence was obtained by cross-referencing the study participants with the extensive Taiwan Cancer Registry database, spanning from January 1997 to December 2008. The identification of cancer cases entailed a thorough examination of histological discharge forms and oncology reports, wherein classification was performed using the ICD-9 (International Classification of Diseases, Ninth Revision) codes (**Supplementary Table S2**). Follow-up endpoint of cancer incidence was set as December 31, 2008.

### Statistical analysis

Baseline characteristics were described across their survivorship as frequency (n) and proportion (%) for categorical variables and mean (± standard deviation) for normally distributed continuous variables. Prior to conducting risk analyses, the indicators were subjected to log transformation and standardization. This involved calculating the log ratio, subtracting the mean of the log ratio, and normalizing it with the standard deviation of the logarithmic ratio. To evaluate the risk of cancer, hazard ratios (HRs) were estimated, considering a standard deviation increment in each log-transformed indicator. The Cox proportional hazards model was utilized, adjusting for various factors including age at recruitment, age-square, sex, education level, occupation, marital status, BMI, smoking status, drinking status, physical activity, dietary habits, and family history of cancer. To ensure the validity of the analysis, the proportionality of hazards assumption was evaluated using the Schoenfeld residuals method, and the results indicated satisfactory adherence to this assumption (P>0.5). Restricted cubic spline was adopted to explore nonlinear associations between indicators of inflammation and cancer incidence. VanderWeele’s mediation analysis was employed to investigate the potential mediating role of obesity in the relationship between inflammation indicators and the risk of cancer. VanderWeele’s mediation analysis was used to explore whether the cancer risk of inflammation indicators were mediated by affecting obesity (34).

Sensitivity analyses were conducted including: analysis using non-log transformation data; and analysis excluding participants with incomplete covariate data.

All statistical analyses were conducted using two-sided tests, and a significance level of P < 0.05 was deemed as statistically significant. To address the possibility of Type I errors resulting from multiple comparisons, p-values were adjusted using the Bonferroni method. The R version 4.1.3 (R Foundation) was utilized for all statistical analyses, unless otherwise specified.

## Results

### Baseline characteristics

We first assessed a total of 457,806 participants at baseline. In all, 356,554 participants were kept in the main analysis after excluding participants under the age of 18, those with prevalent cancer, those with less than one year of follow-up, and those with missing data in CRP, ALB, GLO, PLA, and WBC (**Supplementary Figure S1**). The baseline characteristics of the participants in MJ cohort were displayed in **Table 1**. Over a median follow-up of 8.2 years (interquartile range, 7.1-9.0 years), 9,048 cancer cases were identified according to ICD9 or ICD10 (**Supplementary Table S1**).

**Table 1:** Characteristics of participants in this study (MJ Cohort Study, 1996-2008).

Characteristics	All	Cancer-free participants	Incident cancer cases
	(n=356554)	(n=347506)	(n=9048)
Age (years)	41.9 (14)	41.6 (13.9)	55.7 (13.5)
Sex (%)			
Male	167026 (46.8)	162325 (46.7)	4701 (52)
Female	189528 (53.2)	185181 (53.3)	4347 (48)
Education (%)			
High	81386 (22.8)	80506 (23.2)	880 (9.7)
Median	161455 (45.3)	158795 (45.7)	2660 (29.4)
Low	103056 (28.9)	97945 (28.2)	5111 (56.5)
Missing	10657 (3)	10260 (3)	397 (4.4)
Marital status (%)			
Unmarried	239185 (67.1)	232156 (66.8)	7029 (77.7)
Married	102396 (28.7)	100836 (29)	1560 (17.2)
Missing	14973 (4.2)	14514 (4.2)	459 (5.1)
Smoking status (%)			
Never	232025 (65.1)	227073 (65.3)	4952 (54.7)
Former	21160 (5.9)	20292 (5.8)	868 (9.6)
Current	79279 (22.2)	76943 (22.1)	2336 (25.8)
Missing	24090 (6.8)	23198 (6.7)	892 (9.9)
Alcohol consumption (%)			
Never	251848 (70.6)	246393 (70.9)	5455 (60.3)
Former	10586 (3.0)	10117 (2.9)	469 (5.2)
Current	65336 (18.3)	63208 (18.2)	2128 (23.5)
Missing	28784 (8.1)	27788 (8.0)	996 (11.0)
Physical activity (%)			
Fully active	87255 (24.5)	84667 (24.4)	2588 (28.6)
Inactive	164616 (46.2)	160797 (46.3)	3819 (42.2)
Low	85750 (24.0)	83820 (24.1)	1930 (21.3)
Missing	18933 (5.3)	18222 (5.2)	711 (7.9)
Healthy diet (%)			
No	236708 (66.4)	231629 (66.7)	5079 (56.1)
Yes	85835 (24.1)	83278 (24)	2557 (28.3)
Missing	34011 (9.5)	32599 (9.4)	1412 (15.6)
Family history of cancer (%)			
No	262975 (73.8)	256141 (73.7)	6834 (75.5)
Yes	93579 (26.2)	91365 (26.3)	2214 (24.5)
Anthropometric Measurements			
ABSI,	8 (1.7)	8 (1.8)	8.4 (1.6)
BMI, kg/m2	23.1 (3.6)	23.1 (3.6)	23.9 (3.4)
BF%	26.1 (6.6)	26.1 (6.6)	26 (6.5)
HC, cm	94.3 (5.9)	94.3 (5.9)	94.8 (5.5)
LBM, kg	45 (8.5)	45 (8.5)	45.7 (8.1)
WC, cm	77.1 (9.4)	77 (9.5)	80 (8.7)
WHtR	0.5 (0.1)	0.5 (0.1)	0.5 (0.1)
WHR	0.8 (0.1)	0.8 (0.1)	0.8 (0.1)
Inflammation indicators			
CRP, mg/dl	1 (1-2)	1 (1-3)	1 (1-3)
WBC, 10^3^/μl	6.2 (5.2-7.3)	6.2 (5.2-7.5)	6.3 (5.3-7.5)
MO,10^3^/μl	0.4 (0.4-0.5)	0.4 (0.4-0.6)	0.5 (0.4-0.6)
LY, 10^3^/μl	2.1 (1.7-2.5)	2.1 (1.7-2.6)	2.1 (1.7-2.6)
NE, 10^3^/μl	3.4 (2.7-4.2)	3.4 (2.7-4.3)	3.4 (2.7-4.3)
HEMO, g/dl	14.1 (13.1-15.3)	14.1 (13.1-15)	14 (13-15)
GLO, g/dl	3 (2.8-3.3)	3 (2.8-3.4)	3.1 (2.8-3.4)
ALB, g/dl	4.5 (4.4-4.7)	4.5 (4.4-4.6)	4.4 (4.2-4.6)
PLA, 10^3^/μl	234 (201-271)	234 (202-258)	219 (182-258)

Values are numbers (percentages), means (SDs), medians (interquartile ranges) unless stated otherwise. ABSI, A Body Shape Index; BMI, Body Mass Index; BF%, body fat percentage; HC, Hip Circumference; WC, Waist circumference; WHR, Waist-to-Hip Ratio; WHtR, waist-to-height ratio; CRP, C-reactive Protein; TP, total protein; ALB, albumin; GLO, globulin; PLA, platelets; HEMO, hemoglobin.

### Observational association evaluation

**Table 2** showed the associations between systemic inflammation markers and the incidence of overall cancer, as well as obesity-related and non-obesity-related cancer. For individual biomarkers, the overall cancer risk significantly escalated as the blood CRP (HR=1.036, 95% CI: (1.017-1.054)) and GLO (HR=1.128, 95% CI: (1.105-1.152)) levels increased, and as HEMO (HR=0.863, 95% CI: (0.842-0.884)), ALB (HR=0.846, 95% CI: (0.829-0.863)) and PLA (HR=0.842, 95% CI: (0.827-0.858)) levels decreased. Compared with overall cancer risk, the obesity-related cancer risk showed consistent direction in the above indicators and had a negative association with LY and NE.

**Table 2:** Associations between systemic inflammatory inflammation markers and risk of cancer incidence included in this study (MJ Cohort Study, 1996-2008).

Inflammation markers	Overall cancer		Obesity-related cancer		Non-obesity related cancer	
	Event/PY	9048/2970093	Event/PY	4894/2983029	Event/PY	4154/2986713
	HR (95%CI)	Adjusted *P*	HR (95%CI)	Adjusted *P*	HR (95%CI)	Adjusted *P*
Individual indicators						
CRP	1.036 (1.017-1.054)	**6.93E-03**	1.048 (1.023-1.074)	**8.87E-03**	1.020 (0.994-1.047)	9.90E-01
WBC	0.987 (0.967-1.009)	9.90E-01	0.933 (0.906-0.960)	**1.78E-04**	1.056 (1.024-1.089)	**3.73E-02**
MO	1.031 (1.010-1.054)	2.78E-01	1.034 (1.005-1.065)	9.90E-01	1.029 (0.997-1.061)	9.90E-01
LY	0.968 (0.948-0.988)	1.12E-01	0.950 (0.924-0.977)	**2.19E-02**	0.994 (0.965-1.024)	9.90E-01
NE	0.985 (0.964-1.006)	9.90E-01	0.920 (0.894-0.947)	**8.79E-07**	1.065 (1.032-1.098)	**4.20E-03**
HEMO	0.863 (0.842-0.884)	**8.04E-30**	0.851 (0.824-0.880)	**5.30E-20**	0.890 (0.857-0.924)	**5.93E-08**
GLO	1.128 (1.105-1.152)	**4.70E-29**	1.261 (1.227-1.296)	**4.15E-59**	0.987 (0.958-1.017)	9.90E-01
ALB	0.846 (0.829-0.863)	**5.92E-58**	0.781 (0.761-0.802)	**2.93E-74**	0.944 (0.915-0.973)	**1.42E-02**
PLA	0.842 (0.827-0.858)	**1.63E-70**	0.748 (0.732-0.765)	**3.50E-143**	1.002 (0.973-1.032)	9.90E-01
Composite Indicators						
AGR	0.842 (0.824-0.860)	**1.07E-53**	0.734 (0.713-0.756)	**2.23E-92**	0.990 (0.959-1.021)	9.90E-01
AISI	0.950 (0.931-0.970)	**6.65E-05**	0.868 (0.843-0.892)	**4.53E-21**	1.051 (1.020-1.083)	6.54E-02
CAR	1.035 (1.020-1.050)	**2.11E-04**	1.044 (1.024-1.065)	**7.78E-04**	1.023 (1.001-1.045)	9.90E-01
dNLR	0.968 (0.949-0.988)	1.03E-01	0.916 (0.892-0.941)	**6.17E-09**	1.034 (1.003-1.066)	9.90E-01
HALP	1.063 (1.042-1.085)	**1.87E-07**	1.153 (1.123-1.184)	**4.01E-24**	0.971 (0.942-1.002)	9.90E-01
MLR	1.062 (1.041-1.083)	**1.37E-07**	1.081 (1.052-1.110)	**1.16E-06**	1.037 (1.008-1.068)	8.44E-01
NLPR	1.069 (1.061-1.078)	**3.84E-60**	1.075 (1.066-1.084)	**1.03E-65**	1.045 (1.023-1.067)	**2.98E-03**
NLR	1.018 (0.998-1.039)	9.90E-01	0.978 (0.950-1.006)	9.90E-01	1.059 (1.029-1.090)	**6.49E-03**
PLR	0.900 (0.881-0.918)	**2.77E-22**	0.814 (0.791-0.836)	**3.19E-46**	1.007 (0.977-1.038)	9.90E-01
PNI	0.885 (0.867-0.903)	**4.32E-30**	0.825 (0.803-0.849)	**7.75E-40**	0.968 (0.940-0.998)	9.90E-01
SII	0.919 (0.901-0.938)	**2.30E-14**	0.817 (0.795-0.840)	**2.27E-44**	1.047 (1.017-1.079)	1.23E-01
SIRI	1.036 (1.015-1.057)	**4.28E-02**	1.008 (0.979-1.037)	9.90E-01	1.063 (1.033-1.094)	**2.13E-03**

ALB, albumin; CRP, C-reactive Protein; TP, total protein; GLO, globulin; HEMO, hemoglobin; LY, lymphocyte; MO, monocyte; NE, neutrophil; PLA, platelets; WBC, white blood cells; AGR, Albumin/Globulin ratio; AISI, aggregate index of systemic inflammation; CAR, C-reactive Protein/Albumin; dNLR, derived NLR; HALP, The hemoglobin, albumin, lymphocyte, and platelet score; MLR, monocyte-lymphocyte ratio; NLPR, neutrophil to lymphocyte × platelet ratio; NLR, neutrophil-to-lymphocyte ratio; PLR, platelet-to-lymphocyte ratio; PNI, Prognostic Nutritional Index; SII, systemic immune-inflammation index; SIRI, systemic inflammation response index; PY, person year; HR, Hazard ratios.The bold p values refer to values less than 0.05.

Most of the composite indicators showed a significant relationship to overall cancer risk (except dNLR and NLR) and to obesity-related cancer risk (except NLR and SIRI) (**Table 2**). There was a reduction of association with non-obesity related cancer risk. Interestingly, the level of WBC and its ingredients did not show any statistical association with cancer incidence, whereas the composite indicators composed of them (e.g., AISI, SII, SIRI) all presented a strong association with cancer incidence. These results demonstrated that composite metrics were more sensitive biomarkers on inflammatory status and cancer incidence.

**Figure 1:**
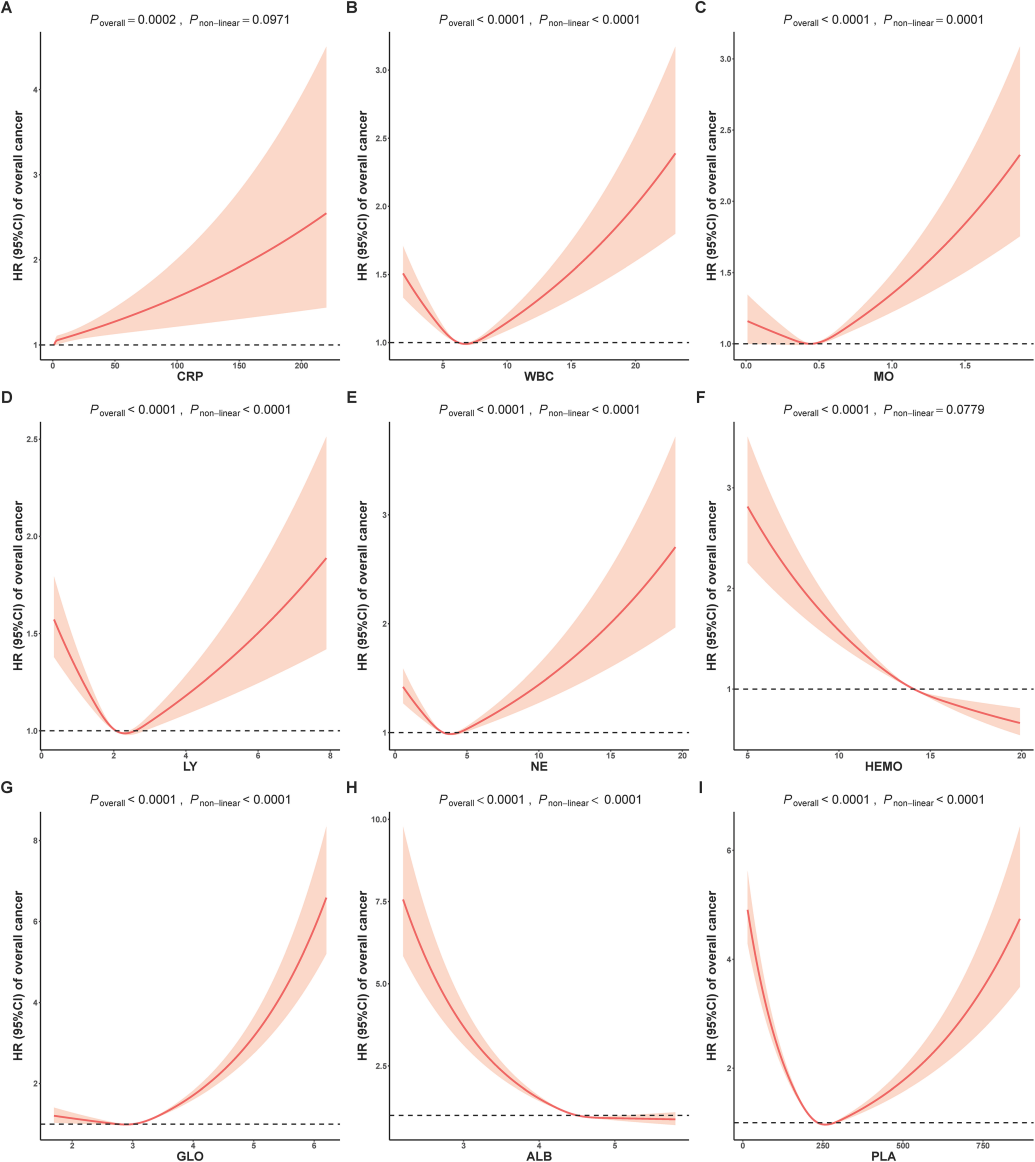
Analysis of the shape of the relationship between individual inflammatory biomarkers of **(A)** CRP, **(B)** WBC, **(C)** MO, **(D)** LY, **(E)** NE, **(F)** HEMO, **(G)** GLO, **(H)** ALB and **(I)** PLA and overall cancer risk using restricted cubic spline. ALB, albumin; CRP, C-reactive Protein; GLO, globulin; HEMO, hemoglobin; LY, lymphocyte; MO, monocyte; NE, neutrophil; PLA, platelets; WBC, white blood cells; HR, Hazard ratios.

**Figure 2:**
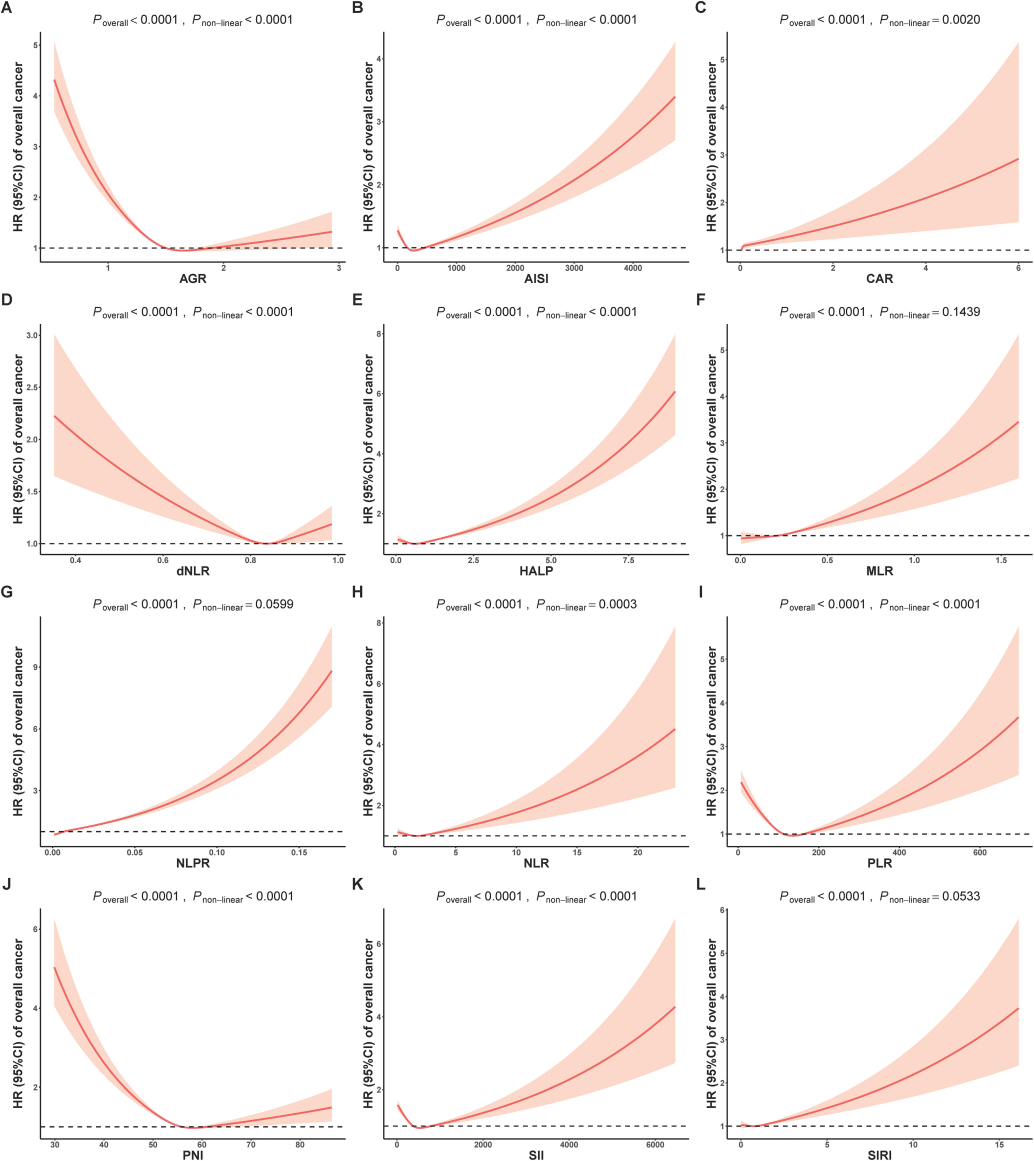
Analysis of the shape of the relationship between composite inflammatory biomarkers of **(A)** AGR, **(B)** AISI, **(C)** CAR, **(D)** dNLR, **(E)** HALP, **(F)** MLR, **(G)** NLPR, **(H)** NLR, **(I)** PLR, **(J)** PNI, **(K)** SII and **(L)** SIRI and overall cancer risk using restricted cubic spline. AGR, Albumin/Globulin ratio; AISI, aggregate index of systemic inflammation; CAR, C-reactive Protein/Albumin; dNLR, derived NLR; HALP, The hemoglobin, albumin, lymphocyte, and platelet score; MLR, monocyte-lymphocyte ratio; NLPR, neutrophil to lymphocyte × platelet ratio; NLR, neutrophil-to-lymphocyte ratio; PLR, platelet-to-lymphocyte ratio; PNI, Prognostic Nutritional Index; SII, systemic immune-inflammation index; SIRI, systemic inflammation response index; HR, Hazard ratios.

To investigate the possibility of non-linear relationships between composite indicators and cancer risk, we conducted an analysis using restricted cubic splines to estimate the associations. In **Figure 1**, almost all individual biomarkers (except CRP and HEMO) displayed non-linear associations with cancer incidence (bonferroni adjusted P non-linear < 0.05). **Figure 2** showed non-linear associations between AGR, AISI, CAR, dNLR, NLR, PLR, PNI and SII and overall cancer risks (bonferroni adjusted P overall < 0.05, and bonferroni adjusted P non-linear < 0.05). In the **Supplementary Figure S2**, we observed distinct non-linear association patterns with the increasing concentration of CRP for obesity-related cancer risk (overall bonferroni-adjusted P < 0.05, and non-linear bonferroni-adjusted P < 0.05)., including a “low-to-fat increase” in risks for the CAR indicator, a “decrease-to-increase” risk pattern for the AISI and HALP indicators, and a “decrease-to-platform” risk pattern for the AGR, dNLR, and PNI indicators. We also found the following positive linear correlation between indicators of MLR, NLPR and SIRI and obesity-related cancer risks (overall bonferroni-adjusted P < 0.05, and non-linear bonferroni-adjusted P < 0.05). Notably, we found that most of inflammatory markers were weaker associated with non-obesity-related cancers than obesity-related cancers (**Supplementary Figures S2–S5**).

### Mediation analysis

**Figure 3:**
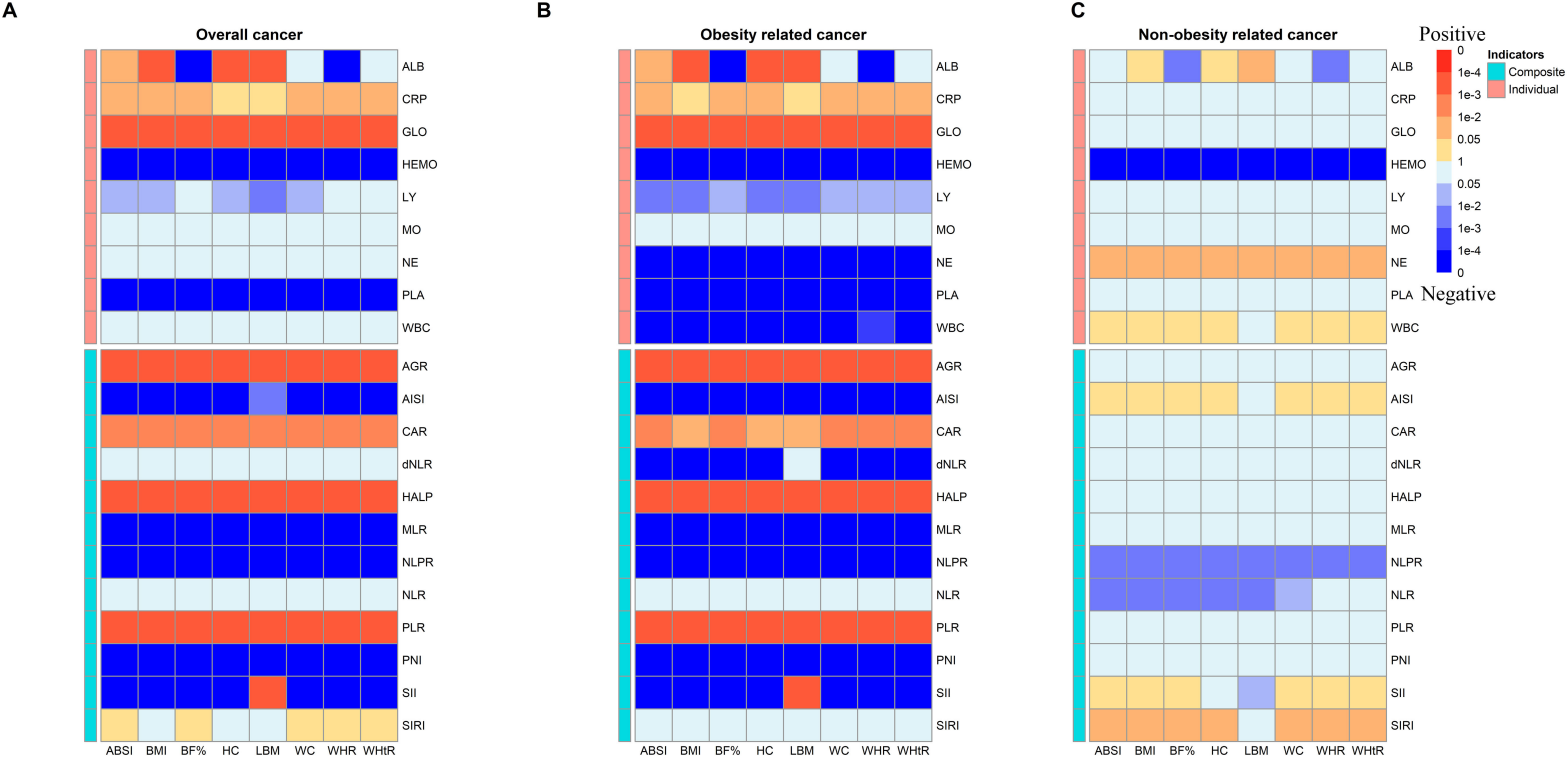
Heatmap of p values for indirect effect of mediation analysis of systemic inflammatory markers in the associations between anthropometric measurements and risk of **(A)** overall cancer incidence, **(B)** obesity related cancer incidence and **(C)** non-obesity related cancer incidence among participants (MJ Cohort Study, 1996-2008). Red blocks indicate a p with positive correlation and blue indicates a p with negative correlation. ALB, albumin; CRP, C-reactive Protein; TP, total protein; GLO, globulin; HEMO, hemoglobin; LY, lymphocyte; MO, monocyte; NE, neutrophil; PLA, platelets; WBC, white blood cells; AGR, Albumin/Globulin ratio; AISI, aggregate index of systemic inflammation; CAR, C-reactive Protein/Albumin; dNLR, derived NLR; HALP, The hemoglobin, albumin, lymphocyte, and platelet score; MLR, monocyte-lymphocyte ratio; NLPR, neutrophil to lymphocyte × platelet ratio; NLR, neutrophil-to-lymphocyte ratio; PLR, platelet-to-lymphocyte ratio; PNI, Prognostic Nutritional Index; SII, systemic immune-inflammation index; SIRI, systemic inflammation response index.

Additionally, we performed formal mediation analyses to quantify the direct effects of anthropometric measurements (ABSI, BMI, BF%, HC, LBM, WC, WHR, and WHtR) on cancer incidence, as well as the indirect effects mediated through various inflammation biomarkers. **Figure 3A** showed that most of indicators mediated the association between anthropometric measurements (e.g., BMI, fat) and overall cancer risk. Further analysis of the mediating utility of inflammation metrics in anthropometric associations with cancer incidence revealed a more significant mediating effect of inflammation in obesity-related cancer than that in non-obesity-related cancer (**Figures 3B, C**). All mediation results were shown **Supplementary Tables S4–S6**. For instance, CAR (HRindirect = 1.0009; 95% CI 1.0005–1.0014; proportion mediated, 29.00%) significantly mediated BMI with the association of overall cancer risk (**Supplementary Table S4**).

## Discussion

In this prospective cohort study comprising 356,654 participants, we observed a significant association between an elevated systemic inflammatory state and increased cancer risks. Additionally, our findings indicated that inflammation biomarkers partially mediated the relationship between obesity and cancer. Our findings highlighted chronic inflammation as a fundamental disorder implicated in cancer development. Moreover, the study demonstrates the feasibility of utilizing inflammation indicators as cost-effective circulating biomarkers for cancer detection.

In our study, we comprehensively exploited individual and composite inflammation indicators as the measure of systemic inflammation. Our study suggested that composite metrics may provide a more comprehensive measure of inflammatory status in participants. Our study results are in general agreement with a previous investigation conducted in the UK Biobank, which similarly identified positive associations between SII and the risks of colorectal and lung cancer, as well as associations between NLR and PLR with these cancer risks. Additionally, negative associations were observed between LMR and the risks of various cancers, particularly colorectal and lung cancer (7). However, NLR in our study was only observed to have correlation with non-obesity related cancers. Furthermore, extensive researches have been conducted to explore the correlation between NLR and cancer prognosis, consistently demonstrating its strong prognostic predictive value (35–37). Also, our study firstly revealed several markers (e.g., HALP, NLPR) were associated with overall or obesity-related cancer incidence.

In a prior meta-analysis, the relationship between inflammation levels, as indicated by CRP, and the overall risk of cancer was thoroughly examined (38). This comprehensive analysis sought to investigate the potential association between inflammatory processes and the development of various types of cancer. In this meta-analysis, a total of 11 prospective studies were included, comprising study populations from developed countries characterized by a relatively elevated mean BMI ranging from 25.9 to 28.9 kg/m2. It is important to note that the presence of obesity within these populations might have an influence on the obtained results. Several studies have investigated the mediating role of inflammation in the relationship between obesity and specific types of cancer (39–42). These studies exhibited conflicting results, and only focused on specific cancer types rather than pan-cancer. In our study, we took obesity status as a confounding factor to explore the relationship between inflammation and cancer risk. It was found that overall cancer risk was positively associated with many inflammation biomarkers (like CRP, GLO, CAR, AISI, etc.), regardless of obesity status. And to further explore the existence of the mediating role of obesity, we performed a mediation analysis of the inflammation-mediated obesity-cancer association. The results identified inflammation as a potential mediator of the obesity-cancer associations. A consistent body of research has repeatedly shown a strong association between visceral adipose tissue, a type of fat characterized by high metabolic activity, and the development of diverse cancer types. This type of adipose tissue is associated with the release of proinflammatory cytokines, further contributing to the inflammatory milieu associated with cancer development (43, 44). Furthermore, chronic inflammation activates inflammatory signaling pathways, leading to the production of reactive oxygen species and the upregulation of cell proliferation (45). These processes contribute to the progression from incipient neoplasia to the development of cancer (1). The present study further validated these findings prospectively. Understanding the link between inflammation and cancer risk can provide valuable insights into potential preventive and therapeutic strategies.

Chronic inflammation is a well-known mediator of cancer and a core feature of obesity, leading to many complications. Beyond obesity itself, the inflammation caused by obesity introduces additional cancer risks. Adipose tissue can secrete over 50 different adipokines, cytokines, and chemokines, positioning it at the intersection of metabolism and immunity (9, 11). The excessive expansion of adipose tissue during obesity fundamentally alters its histology and function. As fat cell size increases, some cells undergo apoptosis and are surrounded by macrophages, forming crown-like structures, a hallmark of adipose inflammation. Throughout various stages of this process, interactions between adipocytes and resident immune cells enhance lipid breakdown and secretion by adipocytes, along with the production of various pro-inflammatory factors by both adipocytes and immune cells (9). Chronic inflammation as a precursor to cancer development has been observed in various cancer types (2). Chronic inflammation promotes mutation and the proliferation of mutated cells partly by generating harmful reactive oxygen species; it activates transcription factors like NF-κB, STAT3, and AP-1, which enhance cell proliferation and survival, and promotes angiogenesis under hypoxic conditions (12). Furthermore, inflammation facilitates several steps in the metastatic process, a primary mechanism of cancer mortality. These steps, depending on the tumor, include epithelial-mesenchymal transition, intravasation into blood and lymphatic vessels, and seeding and proliferation in new areas through interactions with immune and stromal cells.

This study possesses several strengths that enhance its validity. Firstly, it benefits from a relatively large sample size, providing robust statistical power. Additionally, its prospective design minimizes the potential for recall bias and reverse causality. Furthermore, the utilization of a case-cohort design helps mitigate selection bias, making the findings more reliable when assessing the risk of overall cancer. This study is subject to several limitations that should be acknowledged. Firstly, our analysis did not account for the use of anti-inflammatory drugs or consider the presence of viral or bacterial infections, which could potentially influence the results. Secondly, despite efforts to control for confounding variables, there remains the possibility of unmeasured or unknown confounders affecting the observed associations, such as genetic predisposition, or unmeasured variables. Lastly, due to limited cases of site-specific cancers, we were only able to investigate the association between inflammation indicators and overall cancer risk, without examining specific cancer types. Further research, including diverse cohorts and long-term follow-up, is warranted to validate the findings and elucidate the underlying mechanisms linking systemic inflammation, anthropometric measurements, and cancer risk.

In conclusion, from this large population-based prospective healthy cohort study, we gave the first prospective assessment of the association between diverse composite inflammation markers and pan-cancer risk. It emphasized the significance of systemic inflammation in the cancer development and provided the evidence that excessive obesity indirectly affects the risk of cancer through systemic inflammation.

## Data Availability

The Taiwan MJ Cohort is available to the worldwide research community and offers collaboration. Applicants for data access should contact the MJ Health Research Foundation at [http://www.mjhrf.org/]. Further inquiries can be directed to the corresponding author/s.
