# Supplementary material for: Association of the systemic inflammation and anthropometric measurements with cancer risk: a prospective study in MJ cohort
Source: Front Oncol. 2024 Sep 9;14:1400893. doi: 10.3389/fonc.2024.1400893 (PMC11417304; doi:10.3389/fonc.2024.1400893)
Supplement: Supplementary file 1 [file DataSheet1.docx]

**Table S1. Formula of inflammation markers**

| Inflammation markers | Formula |
| --- | --- |
| aggregate index of systemic inflammation (AISI) | (neutrophils × monocytes × platelets)/lymphocytes |
| systemic immune-inflammation index (SII) | (platelets × neutrophils)/lymphocytes |
| monocyte-lymphocyte ratio (MLR) | monocytes/lymphocytes, |
| neutrophil to lymphocyte × platelet ratio (NLPR) | neutrophils/ (lymphocytes × platelets) |
| neutrophil-to-lymphocyte ratio (NLR) | neutrophils/lymphocytes |
| derived NLR (dNLR) | neutrophils/ (white blood cell-counts-neutrophils) |
| platelet-to-lymphocyte ratio (PLR) | platelets/lymphocytes |
| systemic inflammation response index (SIRI) | neutrophils × monocytes/lymphocytes |
| modified Glasgow Prognostic Score (mGPS) | CRP≤10mg/L=0,CRP>10mg/L=1, CRP>10mg/L and albumin <35g/L=2 |
| C-reactive Protein/Albumin (CRP/ALB) | CRP/albumin |
| Albumin/Globulin ratio (AGR) | Albumin/Globulin |
| Prognostic Nutritional Index (PNI) | 10×albumin (g/dL) + 5×lymphocyte counts (10^9^/L) |
| The hemoglobin, albumin, lymphocyte, and platelet (HALP) score | hemoglobin × albumin × lymphocytes / platelets |

**Table S2.** International Classification of Diseases (ICD) codes used for identification of cancer-specific incidence and mortality

| **Cancer site** | **ICD9** | **ICD10** |
| --- | --- | --- |
| Lip, Oral Cavity, Pharynx | 140, 141, 142, 143, 144, 145, 146, 147, 148, 149 | C00, C01, C02, C03, C04, C05, C06, C07, C08, C09, C10, C11, C12, C13, C14 |
| Stomach | 151 | C16 |
| Colon, Rectum | 153, 154 | C18, C19, C20 |
| Liver | 155 | C22 |
| Pancreas | 157 | C25 |
| Lung, Bronchus, Trachea | 162 | C33, C34 |
| Breast | 174 | C50 |
| Cervix Uteri | 180 | C53 |
| Corpus Uteri, NOS | 179, 182 | C54, C55 |
| Ovary | 183 | C56 |
| Prostate | 185 | C61 |
| Kidney | 189 | C64 |
| Bladder | 188 | C67 |
| Thyroid | 193 | C73 |

**Table S3.** Strength of the Evidence for a Cancer-Preventive Effect of the Absence of Excess Body Fatness, According to Cancer Site or Type

| Cancer Site or Type | Strength of the Evidence | Relative Risk of the Highest BMI Category Evaluated versus Normal BMI (95% CI) | ICD9 | ICD10 |
| --- | --- | --- | --- | --- |
| Esophagus: adenocarcinoma | Sufficient | 4.8 (3.0–7.7) | 150 | C15 |
| Gastric cardia | Sufficient | 1.8 (1.3–2.5) | 1510 | C16 |
| Colon and rectum | Sufficient | 1.3 (1.3–1.4) | 153, 154 | C18, C19, C20 |
| Liver | Sufficient | 1.8 (1.6–2.1) | 155 | C22 |
| Gallbladder | Sufficient | 1.3 (1.2–1.4) | 156 | C23 |
| Pancreas | Sufficient | 1.5 (1.2–1.8) | 157 | C25 |
| Breast: postmenopausal | Sufficient | 1.1 (1.1–1.2) | 174 | C50 |
| Corpus uteri | Sufficient | 7.1 (6.3–8.1) | 179, 182 | C54, C55 |
| Ovary | Sufficient | 1.1 (1.1–1.2) | 183 | C56 |
| Kidney: renal-cell | Sufficient | 1.8 (1.7–1.9) | 189 | C64 |
| Meningioma | Sufficient | 1.5 (1.3–1.8) | 1921 | C700 |
| Thyroid | Sufficient | 1.1 (1.0–1.1)§ | 193 | C73 |
| Multiple myeloma | Sufficient | 1.5 (1.2–2.0) | 203 | C90 |
| Male breast cancer | Limited | NA | NA | NA |
| Fatal prostate cancer | Limited | NA | NA | NA |
| Diffuse large B-cell lymphoma | Limited | NA | NA | NA |
| Esophagus: squamous-cell carcinoma | Inadequate | NA | NA | NA |
| Gastric noncardia | Inadequate | NA | NA | NA |
| Extrahepatic biliary tract | Inadequate | NA | NA | NA |
| Lung | Inadequate | NA | NA | NA |
| Skin: cutaneous melanoma | Inadequate | NA | NA | NA |
| Testis | Inadequate | NA | NA | NA |
| Urinary bladder | Inadequate | NA | NA | NA |
| Brain or spinal cord: glioma | Inadequate | NA | NA | NA |

**Table S4.** Mediation analysis of systemic inflammatory markers in the associations between anthropometric measurements and overall cancer incidence among participants (MJ Cohort Study, 1996-2008)

| Indirect effect | ABSI | | | BMI | | | BF% | | | HC | | |
| --- | --- | --- | --- | --- | --- | --- | --- | --- | --- | --- | --- | --- |
|  | HR (95%CI) | proportion | Adjust *P* | HR (95%CI) | proportion | Adjust *P* | HR (95%CI) | proportion | Adjust *P* | HR (95%CI) | proportion | Adjust *P* |
| Individual indicators |  |  |  |  |  |  |  |  |  |  |  |  |
| CRP | 1.0035 (1.0016-1.0054) | 0.02 | 6.64E-03 | 1.0017 (1.0008-1.0027) | 0.54 | 9.92E-03 | 1.0009 (1.0004-1.0014) | -0.30 | 2.99E-03 | 1.0007 (1.0003-1.0011) | 0.18 | 1.27E-02 |
| WBC | 0.9984 (0.9961-1.0007) | -0.02 | 1.00E+00 | 0.9991 (0.9979-1.0002) | -0.33 | 1.00E+00 | 0.9996 (0.9990-1.0003) | 0.11 | 1.00E+00 | 0.9996 (0.9992-1.0001) | -0.10 | 1.00E+00 |
| MO | 1.0018 (1.0003-1.0033) | 0.02 | 3.33E-01 | 1.0009 (1.0001-1.0016) | 0.29 | 4.26E-01 | 1.0005 (1.0001-1.0009) | -0.17 | 1.87E-01 | 1.0003 (1.0000-1.0006) | 0.09 | 4.88E-01 |
| LY | 0.9965 (0.9943-0.9987) | -0.04 | 3.45E-02 | 0.9980 (0.9968-0.9992) | -0.66 | 1.71E-02 | 0.9991 (0.9985-0.9997) | 0.27 | 9.92E-02 | 0.9992 (0.9987-0.9997) | -0.21 | 1.55E-02 |
| NE | 0.9986 (0.9969-1.0003) | -0.02 | 1.00E+00 | 0.9993 (0.9984-1.0001) | -0.27 | 1.00E+00 | 0.9997 (0.9992-1.0001) | 0.10 | 1.00E+00 | 0.9997 (0.9994-1.0000) | -0.08 | 1.00E+00 |
| HEMO | 0.9917 (0.9902-0.9932) | -0.02 | 0.00E+00 | 0.9956 (0.9949-0.9964) | -0.89 | 0.00E+00 | 0.9969 (0.9963-0.9975) | 0.01 | 0.00E+00 | 0.9982 (0.9979-0.9985) | -0.39 | 0.00E+00 |
| GLO | 1.0041 (1.0034-1.0049) | -0.09 | 0.00E+00 | 1.0018 (1.0014-1.0021) | 0.53 | 0.00E+00 | 1.0013 (1.0011-1.0015) | -0.42 | 0.00E+00 | 1.0008 (1.0007-1.0010) | 0.22 | 0.00E+00 |
| ALB | 1.0006 (1.0003-1.0010) | 0.60 | 9.93E-03 | 1.0005 (1.0003-1.0007) | 0.09 | 1.02E-07 | 0.9986 (0.9985-0.9988) | 0.69 | 0.00E+00 | 1.0004 (1.0003-1.0005) | 0.09 | 2.23E-11 |
| PLA | 0.9893 (0.9880-0.9905) | 0.03 | 0.00E+00 | 0.9938 (0.9930-0.9945) | -1.68 | 0.00E+00 | 0.9960 (0.9955-0.9964) | 0.87 | 0.00E+00 | 0.9981 (0.9979-0.9983) | -0.74 | 0.00E+00 |
| Composite Indicators |  |  |  |  |  |  |  |  |  |  |  |  |
| AGR | 1.0054 (1.0047-1.0062) | -0.09 | 0.00E+00 | 1.0024 (1.0021-1.0028) | 0.55 | 0.00E+00 | 1.0010 (1.0008-1.0011) | -0.38 | 0.00E+00 | 1.0012 (1.0010-1.0014) | 0.30 | 0.00E+00 |
| AISI | 0.9971 (0.9960-0.9983) | 0.29 | 1.71E-05 | 0.9985 (0.9979-0.9991) | -0.66 | 1.39E-05 | 0.9991 (0.9987-0.9995) | 0.25 | 2.88E-05 | 0.9995 (0.9993-0.9997) | -0.14 | 1.76E-05 |
| CAR | 1.0019 (1.0011-1.0027) | 0.01 | 2.31E-04 | 1.0009 (1.0005-1.0014) | 0.29 | 2.83E-04 | 1.0005 (1.0003-1.0007) | -0.15 | 1.55E-04 | 1.0004 (1.0002-1.0006) | 0.10 | 3.25E-04 |
| dNLR | 0.9996 (0.9993-0.9999) | -0.93 | 1.02E-01 | 0.9998 (0.9996-0.9999) | -0.09 | 9.62E-02 | 0.9999 (0.9998-1.0000) | 0.04 | 1.01E-01 | 0.9999 (0.9999-1.0000) | -0.02 | 1.44E-01 |
| HALP | 1.0031 (1.0020-1.0042) | -0.16 | 6.17E-07 | 1.0016 (1.0010-1.0021) | 0.86 | 1.06E-06 | 1.0009 (1.0006-1.0012) | -0.23 | 1.26E-07 | 1.0007 (1.0004-1.0010) | 0.21 | 2.02E-06 |
| MLR | 0.9984 (0.9979-0.9990) | -0.02 | 9.36E-07 | 0.9990 (0.9987-0.9994) | -0.31 | 4.54E-07 | 0.9996 (0.9994-0.9997) | 0.14 | 1.46E-06 | 0.9996 (0.9995-0.9997) | -0.10 | 6.33E-07 |
| NLPR | 0.9975 (0.9972-0.9979) | -0.02 | 0.00E+00 | 0.9985 (0.9983-0.9987) | -0.49 | 0.00E+00 | 0.9991 (0.9990-0.9992) | 0.28 | 0.00E+00 | 0.9994 (0.9994-0.9995) | -0.15 | 0.00E+00 |
| NLR | 0.9998 (0.9996-1.0000) | -0.15 | 1.00E+00 | 0.9999 (0.9997-1.0000) | -0.04 | 1.00E+00 | 1.0000 (0.9999-1.0000) | 0.01 | 1.00E+00 | 0.9999 (0.9999-1.0000) | -0.02 | 1.00E+00 |
| PLR | 1.0047 (1.0037-1.0056) | -0.09 | 0.00E+00 | 1.0022 (1.0018-1.0027) | 0.02 | 0.00E+00 | 1.0008 (1.0006-1.0010) | -0.20 | 0.00E+00 | 1.0011 (1.0009-1.0014) | 0.35 | 0.00E+00 |
| PNI | 0.9910 (0.9896-0.9925) | -0.15 | 0.00E+00 | 0.9952 (0.9944-0.9960) | -0.01 | 0.00E+00 | 0.9969 (0.9963-0.9974) | 0.01 | 0.00E+00 | 0.9982 (0.9979-0.9985) | -0.45 | 0.00E+00 |
| SII | 0.9979 (0.9973-0.9984) | 0.02 | 8.02E-13 | 0.9988 (0.9986-0.9991) | -0.76 | 3.78E-13 | 0.9991 (0.9989-0.9993) | 0.22 | 1.49E-13 | 0.9997 (0.9997-0.9998) | -0.08 | 2.09E-09 |
| SIRI | 1.0009 (1.0003-1.0015) | 0.70 | 4.81E-02 | 1.0004 (1.0001-1.0007) | 0.12 | 5.02E-02 | 1.0003 (1.0001-1.0004) | -0.08 | 4.17E-02 | 1.0001 (1.0000-1.0002) | 0.04 | 5.59E-02 |
| Indirect effect | LBM | | | WC | | | WHR | | | WHtR | | |
|  | HR (95%CI) | proportion | Adjust *P* | HR (95%CI) | proportion | Adjust *P* | HR (95%CI) | proportion | Adjust *P* | HR (95%CI) | proportion | Adjust *P* |
| Individual indicators |  |  |  |  |  |  |  |  |  |  |  |  |
| CRP | 1.0005 (1.0002-1.0007) | 0.06 | 2.06E-02 | 1.0006 (1.0003-1.0010) | 0.01 | 7.20E-03 | 1.0752 (1.0347-1.1173) | -0.40 | 4.53E-03 | 1.1204 (1.0558-1.1890) | -0.53 | 3.70E-03 |
| WBC | 0.9998 (0.9996-1.0000) | -0.03 | 1.00E+00 | 0.9997 (0.9992-1.0001) | -0.97 | 1.00E+00 | 0.9648 (0.9122-1.0204) | 0.17 | 1.00E+00 | 0.9508 (0.8760-1.0320) | 0.19 | 1.00E+00 |
| MO | 1.0002 (1.0000-1.0004) | 0.03 | 5.56E-01 | 1.0004 (1.0001-1.0006) | 0.84 | 3.50E-01 | 1.0468 (1.0103-1.0846) | -0.23 | 2.42E-01 | 1.0687 (1.0158-1.1244) | -0.28 | 2.16E-01 |
| LY | 0.9994 (0.9991-0.9998) | -0.07 | 9.40E-03 | 0.9993 (0.9989-0.9997) | -0.02 | 3.24E-02 | 0.9299 (0.8867-0.9752) | 0.33 | 5.74E-02 | 0.8939 (0.8297-0.9631) | 0.41 | 6.69E-02 |
| NE | 0.9999 (0.9997-1.0000) | -0.02 | 1.00E+00 | 0.9997 (0.9994-1.0001) | -0.86 | 1.00E+00 | 0.9665 (0.9247-1.0103) | 0.16 | 1.00E+00 | 0.9538 (0.8957-1.0156) | 0.18 | 1.00E+00 |
| HEMO | 0.9989 (0.9987-0.9991) | -0.13 | 0.00E+00 | 0.9984 (0.9982-0.9987) | -0.02 | 0.00E+00 | 0.8445 (0.8186-0.8712) | 0.01 | 0.00E+00 | 0.7877 (0.7541-0.8228) | 0.01 | 0.00E+00 |
| GLO | 1.0002 (1.0001-1.0003) | 0.02 | 3.10E-05 | 1.0008 (1.0007-1.0010) | 22.20 | 0.00E+00 | 1.1074 (1.0868-1.1283) | -0.36 | 0.00E+00 | 1.1569 (1.1273-1.1873) | -0.46 | 0.00E+00 |
| ALB | 1.0010 (1.0008-1.0011) | 0.11 | 0.00E+00 | 1.0000 (0.9999-1.0000) | 0.68 | 1.00E+00 | 0.9602 (0.9499-0.9707) | 0.12 | 4.34E-12 | 1.0081 (0.9972-1.0191) | -0.02 | 1.00E+00 |
| PLA | 0.9997 (0.9996-0.9998) | -0.05 | 4.59E-09 | 0.9980 (0.9977-0.9982) | 0.06 | 0.00E+00 | 0.7569 (0.7324-0.7823) | 0.01 | 0.00E+00 | 0.6695 (0.6390-0.7015) | 0.01 | 0.00E+00 |
| Composite Indicators |  |  |  |  |  |  |  |  |  |  |  |  |
| AGR | 1.0007 (1.0006-1.0008) | 0.08 | 0.00E+00 | 1.0010 (1.0009-1.0012) | -0.04 | 0.00E+00 | 1.1146 (1.0962-1.1333) | -0.31 | 0.00E+00 | 1.1979 (1.1686-1.2280) | -0.48 | 0.00E+00 |
| AISI | 0.9999 (0.9999-1.0000) | -0.01 | 7.83E-03 | 0.9994 (0.9992-0.9996) | -0.02 | 1.56E-05 | 0.9216 (0.8916-0.9525) | 0.38 | 2.62E-05 | 0.8969 (0.8583-0.9373) | 0.40 | 2.64E-05 |
| CAR | 1.0002 (1.0001-1.0003) | 0.03 | 4.34E-04 | 1.0004 (1.0002-1.0005) | 0.70 | 2.42E-04 | 1.0427 (1.0236-1.0622) | -0.22 | 1.89E-04 | 1.0643 (1.0356-1.0937) | -0.28 | 1.66E-04 |
| dNLR | 1.0000 (1.0000-1.0000) | 0.00 | 1.00E+00 | 0.9999 (0.9998-1.0000) | -0.29 | 1.00E-01 | 0.9859 (0.9763-0.9957) | 0.07 | 9.98E-02 | 0.9800 (0.9665-0.9938) | 0.08 | 9.45E-02 |
| HALP | 1.0006 (1.0004-1.0009) | 0.08 | 8.86E-06 | 1.0006 (1.0004-1.0008) | 0.02 | 7.13E-07 | 1.0592 (1.0378-1.0810) | -0.32 | 6.57E-07 | 1.1009 (1.0647-1.1383) | -0.40 | 3.68E-07 |
| MLR | 0.9997 (0.9995-0.9998) | -0.04 | 6.00E-07 | 0.9997 (0.9996-0.9998) | -0.85 | 1.13E-06 | 0.9786 (0.9706-0.9868) | 0.10 | 7.03E-06 | 0.9528 (0.9363-0.9696) | 0.18 | 1.16E-06 |
| NLPR | 0.9996 (0.9996-0.9997) | -0.05 | 0.00E+00 | 0.9996 (0.9995-0.9996) | -0.01 | 0.00E+00 | 0.9603 (0.9542-0.9664) | 0.20 | 0.00E+00 | 0.9267 (0.9171-0.9363) | 0.30 | 0.00E+00 |
| NLR | 0.9999 (0.9998-1.0000) | -0.01 | 1.00E+00 | 1.0000 (1.0000-1.0000) | -0.05 | 1.00E+00 | 1.0018 (0.9994-1.0042) | -0.01 | 1.00E+00 | 0.9981 (0.9956-1.0007) | 0.01 | 1.00E+00 |
| PLR | 1.0011 (1.0009-1.0014) | 0.16 | 0.00E+00 | 1.0009 (1.0007-1.0011) | 0.03 | 0.00E+00 | 1.0844 (1.0656-1.1035) | -0.49 | 0.00E+00 | 1.1520 (1.1188-1.1863) | -0.61 | 0.00E+00 |
| PNI | 0.9991 (0.9990-0.9993) | -0.11 | 0.00E+00 | 0.9982 (0.9979-0.9985) | 0.38 | 0.00E+00 | 0.8045 (0.7755-0.8347) | 0.68 | 0.00E+00 | 0.7367 (0.6998-0.7756) | 0.84 | 0.00E+00 |
| SII | 1.0003 (1.0002-1.0004) | 0.04 | 3.45E-09 | 0.9995 (0.9994-0.9997) | -0.04 | 5.08E-13 | 0.9235 (0.9050-0.9424) | 0.36 | 3.12E-13 | 0.9071 (0.8850-0.9298) | 0.33 | 2.14E-13 |
| SIRI | 1.0000 (1.0000-1.0000) | 0.00 | 1.00E+00 | 1.0002 (1.0001-1.0003) | 0.45 | 4.86E-02 | 1.0335 (1.0123-1.0550) | -0.17 | 3.79E-02 | 1.0402 (1.0148-1.0663) | -0.17 | 3.80E-02 |

Indirect effect represents indirect effect of mediation analysis of systemic inflammatory markers in the associations between anthropometric measurements and overall cancer incidence

**Table S5.** Mediation analysis of systemic inflammatory markers in the associations between anthropometric measurements and obesity-related cancer incidence among participants (MJ Cohort Study, 1996-2008)

| Indirect effect | ABSI | | | BMI | | | BF% | | | HC | | |
| --- | --- | --- | --- | --- | --- | --- | --- | --- | --- | --- | --- | --- |
|  | HR (95%CI) | proportion | Adjust *P* | HR (95%CI) | proportion | Adjust *P* | HR (95%CI) | proportion | Adjust *P* | HR (95%CI) | proportion | Adjust *P* |
| Individual indicators | |  |  |  |  |  |  |  |  |  |  |  |
| CRP | 1.0048 (1.0022-1.0074) | 0.24 | 7.16E-03 | 1.0023 (1.0009-1.0036) | 0.16 | 1.75E-02 | 1.0012 (1.0006-1.0019) | 0.83 | 3.35E-03 | 1.0010 (1.0004-1.0015) | 0.11 | 9.73E-03 |
| WBC | 0.9922 (0.9891-0.9953) | -0.42 | 2.19E-05 | 0.9957 (0.9941-0.9973) | -0.32 | 4.27E-06 | 0.9980 (0.9971-0.9988) | -0.02 | 6.29E-05 | 0.9985 (0.9978-0.9991) | -0.19 | 1.84E-05 |
| MO | 1.0018 (0.9998-1.0038) | 0.09 | 1.00E+00 | 1.0008 (0.9998-1.0018) | 0.06 | 1.00E+00 | 1.0005 (1.0000-1.0011) | 0.40 | 1.00E+00 | 1.0003 (0.9999-1.0007) | 0.04 | 1.00E+00 |
| LY | 0.9946 (0.9916-0.9976) | -0.28 | 8.54E-03 | 0.9968 (0.9952-0.9984) | -0.23 | 1.89E-03 | 0.9986 (0.9978-0.9994) | -0.01 | 1.99E-02 | 0.9988 (0.9982-0.9995) | -0.14 | 6.08E-03 |
| NE | 0.9931 (0.9908-0.9954) | -0.37 | 1.20E-07 | 0.9964 (0.9952-0.9976) | -0.27 | 3.68E-08 | 0.9982 (0.9975-0.9988) | -0.02 | 2.76E-07 | 0.9987 (0.9982-0.9991) | -0.16 | 1.23E-07 |
| HEMO | 0.9911 (0.9891-0.9930) | -0.40 | 0.00E+00 | 0.9952 (0.9942-0.9962) | -0.31 | 0.00E+00 | 0.9966 (0.9959-0.9974) | -0.02 | 0.00E+00 | 0.9981 (0.9977-0.9985) | -0.21 | 0.00E+00 |
| GLO | 1.0080 (1.0069-1.0090) | 0.45 | 0.00E+00 | 1.0034 (1.0029-1.0039) | 0.24 | 0.00E+00 | 1.0025 (1.0022-1.0028) | 0.02 | 0.00E+00 | 1.0016 (1.0014-1.0018) | 0.19 | 0.00E+00 |
| ALB | 1.0009 (1.0004-1.0014) | 0.05 | 9.34E-03 | 1.0007 (1.0005-1.0010) | 0.05 | 6.29E-08 | 0.9980 (0.9977-0.9982) | -0.91 | 0.00E+00 | 1.0005 (1.0004-1.0007) | 0.06 | 7.89E-12 |
| PLA | 0.9819 (0.9804-0.9835) | -0.01 | 0.00E+00 | 0.9894 (0.9885-0.9903) | -0.01 | 0.00E+00 | 0.9931 (0.9925-0.9937) | 0.26 | 0.00E+00 | 0.9968 (0.9965-0.9971) | -0.45 | 0.00E+00 |
| Composite Indicators |  |  |  |  |  |  |  |  |  |  |  |  |
| AGR | 1.0097 (1.0086-1.0108) | 0.56 | 0.00E+00 | 1.0043 (1.0038-1.0048) | 0.28 | 0.00E+00 | 1.0017 (1.0015-1.0020) | 0.01 | 0.00E+00 | 1.0021 (1.0019-1.0024) | 0.26 | 0.00E+00 |
| AISI | 0.9921 (0.9906-0.9937) | -0.45 | 0.00E+00 | 0.9960 (0.9952-0.9968) | -0.33 | 0.00E+00 | 0.9975 (0.9970-0.9980) | -0.05 | 0.00E+00 | 0.9987 (0.9984-0.9989) | -0.17 | 0.00E+00 |
| CAR | 1.0024 (1.0012-1.0035) | 0.12 | 8.81E-04 | 1.0012 (1.0006-1.0017) | 0.08 | 1.39E-03 | 1.0006 (1.0003-1.0009) | 0.41 | 5.92E-04 | 1.0005 (1.0002-1.0007) | 0.06 | 1.00E-03 |
| dNLR | 0.9988 (0.9983-0.9992) | -0.07 | 4.18E-07 | 0.9993 (0.9991-0.9995) | -0.05 | 1.49E-07 | 0.9996 (0.9995-0.9997) | -0.36 | 1.83E-07 | 0.9998 (0.9997-0.9999) | -0.02 | 1.85E-05 |
| HALP | 1.0074 (1.0060-1.0089) | 0.43 | 0.00E+00 | 1.0037 (1.0030-1.0045) | 0.32 | 0.00E+00 | 1.0020 (1.0016-1.0024) | 0.04 | 0.00E+00 | 1.0017 (1.0014-1.0021) | 0.22 | 0.00E+00 |
| MLR | 0.9981 (0.9973-0.9988) | -0.10 | 1.21E-05 | 0.9988 (0.9983-0.9992) | -0.09 | 4.21E-06 | 0.9994 (0.9992-0.9997) | -0.39 | 1.49E-05 | 0.9995 (0.9993-0.9997) | -0.06 | 9.37E-06 |
| NLPR | 0.9973 (0.9970-0.9977) | -0.14 | 0.00E+00 | 0.9984 (0.9982-0.9986) | -0.12 | 0.00E+00 | 0.9990 (0.9989-0.9992) | -0.66 | 0.00E+00 | 0.9994 (0.9993-0.9995) | -0.07 | 0.00E+00 |
| NLR | 1.0003 (1.0000-1.0006) | 0.01 | 1.00E+00 | 1.0002 (1.0000-1.0004) | 0.01 | 1.00E+00 | 1.0001 (1.0000-1.0002) | 0.06 | 1.00E+00 | 1.0001 (1.0000-1.0002) | 0.01 | 1.00E+00 |
| PLR | 1.0092 (1.0078-1.0106) | 0.54 | 0.00E+00 | 1.0044 (1.0037-1.0050) | 0.38 | 0.00E+00 | 1.0016 (1.0013-1.0018) | 0.04 | 0.00E+00 | 1.0022 (1.0019-1.0026) | 0.29 | 0.00E+00 |
| PNI | 0.9861 (0.9841-0.9881) | -0.73 | 0.00E+00 | 0.9924 (0.9914-0.9935) | -0.49 | 0.00E+00 | 0.9950 (0.9943-0.9958) | -0.03 | 0.00E+00 | 0.9972 (0.9968-0.9976) | -0.33 | 0.00E+00 |
| SII | 0.9949 (0.9940-0.9957) | -0.33 | 0.00E+00 | 0.9972 (0.9968-0.9977) | -0.25 | 0.00E+00 | 0.9979 (0.9976-0.9982) | -0.76 | 0.00E+00 | 0.9994 (0.9992-0.9995) | -0.08 | 0.00E+00 |
| SIRI | 1.0000 (0.9992-1.0009) | 0.00 | 1.00E+00 | 1.0000 (0.9997-1.0004) | 0.00 | 1.00E+00 | 1.0000 (0.9998-1.0002) | 0.01 | 1.00E+00 | 1.0000 (0.9999-1.0001) | 0.00 | 1.00E+00 |
| Indirect effect | LBM | | | WC | | | WHR | | | WHtR | | |
|  | HR (95%CI) | proportion | Adjust *P* | HR (95%CI) | proportion | Adjust *P* | HR (95%CI) | proportion | Adjust *P* | HR (95%CI) | proportion | Adjust *P* |
| Individual indicators | |  |  |  |  |  |  |  |  |  |  |  |
| CRP | 1.0007 (1.0003-1.0010) | 0.06 | 1.08E-02 | 1.0009 (1.0004-1.0014) | 0.26 | 6.16E-03 | 1.1079 (1.0514-1.1675) | -0.16 | 2.63E-03 | 1.1669 (1.0759-1.2655) | 0.52 | 4.07E-03 |
| WBC | 0.9992 (0.9989-0.9995) | -0.08 | 3.32E-05 | 0.9985 (0.9979-0.9991) | -0.51 | 2.59E-05 | 0.8366 (0.7748-0.9034) | 0.03 | 1.12E-04 | 0.7623 (0.6813-0.8529) | -0.01 | 4.59E-05 |
| MO | 1.0002 (1.0000-1.0004) | 0.02 | 1.00E+00 | 1.0004 (1.0000-1.0008) | 0.11 | 1.00E+00 | 1.0499 (1.0004-1.1020) | -0.02 | 1.00E+00 | 1.0681 (0.9967-1.1446) | 0.25 | 1.00E+00 |
| LY | 0.9992 (0.9988-0.9996) | -0.08 | 6.14E-03 | 0.9990 (0.9984-0.9996) | -0.32 | 1.08E-02 | 0.9001 (0.8434-0.9606) | 0.02 | 3.18E-02 | 0.8403 (0.7589-0.9306) | -0.77 | 1.74E-02 |
| NE | 0.9994 (0.9992-0.9996) | -0.05 | 5.09E-07 | 0.9986 (0.9982-0.9991) | -0.46 | 1.28E-07 | 0.8412 (0.7919-0.8936) | 0.02 | 4.31E-07 | 0.7780 (0.7142-0.8476) | -0.01 | 1.95E-07 |
| HEMO | 0.9989 (0.9986-0.9991) | -0.10 | 0.00E+00 | 0.9983 (0.9980-0.9987) | -0.46 | 0.00E+00 | 0.8365 (0.8026-0.8719) | 0.31 | 0.00E+00 | 0.7737 (0.7300-0.8200) | -0.85 | 0.00E+00 |
| GLO | 1.0004 (1.0002-1.0005) | 0.03 | 9.20E-06 | 1.0016 (1.0014-1.0018) | 0.60 | 0.00E+00 | 1.2151 (1.1828-1.2483) | -0.01 | 0.00E+00 | 1.3192 (1.2719-1.3683) | 0.02 | 0.00E+00 |
| ALB | 1.0014 (1.0012-1.0016) | 0.12 | 0.00E+00 | 0.9999 (0.9998-1.0000) | -0.02 | 1.00E+00 | 0.9423 (0.9278-0.9570) | 0.32 | 1.36E-12 | 1.0119 (0.9959-1.0281) | 0.14 | 1.00E+00 |
| PLA | 0.9994 (0.9993-0.9996) | -0.06 | 1.03E-09 | 0.9966 (0.9963-0.9969) | -0.02 | 0.00E+00 | 0.6247 (0.5996-0.6508) | 0.03 | 0.00E+00 | 0.5061 (0.4781-0.5358) | -0.09 | 0.00E+00 |
| Composite Indicators |  |  |  |  |  |  |  |  |  |  |  |  |
| AGR | 1.0013 (1.0010-1.0015) | 0.10 | 0.00E+00 | 1.0018 (1.0016-1.0020) | 0.77 | 0.00E+00 | 1.2129 (1.1824-1.2440) | -0.92 | 0.00E+00 | 1.3776 (1.3287-1.4283) | 0.05 | 0.00E+00 |
| AISI | 0.9998 (0.9997-0.9999) | -0.02 | 9.33E-05 | 0.9984 (0.9981-0.9987) | -0.55 | 0.00E+00 | 0.7984 (0.7627-0.8358) | 0.03 | 0.00E+00 | 0.7383 (0.6948-0.7846) | -0.02 | 0.00E+00 |
| CAR | 1.0003 (1.0002-1.0004) | 0.03 | 1.15E-03 | 1.0005 (1.0002-1.0007) | 0.14 | 8.26E-04 | 1.0548 (1.0289-1.0813) | -0.03 | 5.29E-04 | 1.0814 (1.0423-1.1220) | 0.28 | 6.59E-04 |
| dNLR | 1.0001 (1.0000-1.0001) | 0.01 | 4.32E-01 | 0.9997 (0.9997-0.9998) | -0.08 | 2.96E-07 | 0.9607 (0.9476-0.9740) | 0.74 | 2.33E-07 | 0.9445 (0.9266-0.9626) | -0.26 | 8.84E-08 |
| HALP | 1.0015 (1.0012-1.0019) | 0.16 | 0.00E+00 | 1.0014 (1.0011-1.0017) | 0.46 | 0.00E+00 | 1.1477 (1.1167-1.1795) | -0.73 | 0.00E+00 | 1.2540 (1.1998-1.3107) | 0.80 | 0.00E+00 |
| MLR | 0.9996 (0.9994-0.9997) | -0.04 | 1.02E-05 | 0.9997 (0.9995-0.9998) | -0.10 | 1.64E-05 | 0.9740 (0.9632-0.9849) | 0.56 | 7.34E-05 | 0.9420 (0.9199-0.9647) | -0.25 | 1.73E-05 |
| NLPR | 0.9996 (0.9995-0.9997) | -0.04 | 0.00E+00 | 0.9995 (0.9995-0.9996) | -0.14 | 0.00E+00 | 0.9575 (0.9511-0.9639) | 0.01 | 0.00E+00 | 0.9214 (0.9115-0.9314) | -0.32 | 0.00E+00 |
| NLR | 1.0001 (1.0000-1.0003) | 0.01 | 1.00E+00 | 1.0000 (1.0000-1.0001) | 0.01 | 1.00E+00 | 0.9972 (0.9938-1.0006) | 0.09 | 1.00E+00 | 1.0030 (0.9993-1.0066) | 0.01 | 1.00E+00 |
| PLR | 1.0023 (1.0019-1.0026) | 0.24 | 0.00E+00 | 1.0018 (1.0015-1.0020) | 0.57 | 0.00E+00 | 1.1744 (1.1452-1.2044) | 0.13 | 0.00E+00 | 1.3208 (1.2680-1.3758) | 0.93 | 0.00E+00 |
| PNI | 0.9986 (0.9984-0.9989) | -0.12 | 0.00E+00 | 0.9972 (0.9968-0.9976) | -0.01 | 0.00E+00 | 0.7152 (0.6801-0.7522) | 0.02 | 0.00E+00 | 0.6219 (0.5797-0.6672) | -0.06 | 0.00E+00 |
| SII | 1.0007 (1.0005-1.0008) | 0.07 | 0.00E+00 | 0.9989 (0.9987-0.9991) | -0.41 | 0.00E+00 | 0.8253 (0.8014-0.8499) | 0.02 | 0.00E+00 | 0.7899 (0.7624-0.8184) | -0.01 | 0.00E+00 |
| SIRI | 1.0000 (1.0000-1.0000) | 0.00 | 1.00E+00 | 1.0000 (0.9998-1.0002) | 0.00 | 1.00E+00 | 1.0025 (0.9738-1.0320) | -0.09 | 1.00E+00 | 1.0021 (0.9678-1.0376) | 0.01 | 1.00E+00 |

Indirect effect represents indirect effect of mediation analysis of systemic inflammatory markers in the associations between anthropometric measurements and obesity-related cancer incidence.

**Table S6.** Mediation analysis of systemic inflammatory markers in the associations between anthropometric measurements and non-obesity related cancer incidence among participants (MJ Cohort Study, 1996-2008)

| Indirect effect | ABSI | | | BMI | | | BF% | | | HC | | |
| --- | --- | --- | --- | --- | --- | --- | --- | --- | --- | --- | --- | --- |
|  | HR (95%CI) | proportion | Adjust *P* | HR (95%CI) | proportion | Adjust *P* | HR (95%CI) | proportion | Adjust *P* | HR (95%CI) | proportion | Adjust *P* |
| Individual indicators | |  |  |  |  |  |  |  |  |  |  |  |
| CRP | 1.0019 (0.9992-1.0047) | -0.16 | 1.00E+00 | 1.0010 (0.9996-1.0024) | -0.16 | 1.00E+00 | 1.0005 (0.9998-1.0012) | -0.08 | 1.00E+00 | 1.0004 (0.9998-1.0010) | -0.64 | 1.00E+00 |
| WBC | 1.0056 (1.0022-1.0089) | -0.47 | 2.36E-02 | 1.0029 (1.0012-1.0046) | -0.49 | 1.83E-02 | 1.0016 (1.0007-1.0025) | -0.24 | 1.02E-02 | 1.0010 (1.0004-1.0017) | -0.02 | 3.96E-02 |
| MO | 1.0017 (0.9996-1.0039) | -0.13 | 1.00E+00 | 1.0009 (0.9998-1.0019) | -0.14 | 1.00E+00 | 1.0005 (0.9999-1.0011) | -0.07 | 1.00E+00 | 1.0003 (0.9999-1.0007) | -0.47 | 1.00E+00 |
| LY | 0.9992 (0.9960-1.0025) | 0.06 | 1.00E+00 | 0.9997 (0.9979-1.0014) | 0.05 | 1.00E+00 | 0.9999 (0.9990-1.0008) | 0.01 | 1.00E+00 | 0.9998 (0.9991-1.0005) | 0.31 | 1.00E+00 |
| NE | 1.0049 (1.0024-1.0074) | -0.43 | 2.38E-03 | 1.0025 (1.0012-1.0038) | -0.44 | 2.06E-03 | 1.0014 (1.0007-1.0021) | -0.21 | 1.30E-03 | 1.0009 (1.0004-1.0014) | -0.02 | 3.58E-03 |
| HEMO | 0.9933 (0.9911-0.9955) | 0.69 | 7.88E-08 | 0.9966 (0.9954-0.9977) | 0.77 | 9.04E-08 | 0.9976 (0.9967-0.9984) | 0.39 | 4.06E-07 | 0.9986 (0.9981-0.9990) | -1.55 | 3.35E-08 |
| GLO | 0.9996 (0.9985-1.0006) | 0.04 | 1.00E+00 | 0.9998 (0.9993-1.0002) | 0.03 | 1.00E+00 | 0.9999 (0.9995-1.0002) | 0.02 | 1.00E+00 | 0.9999 (0.9997-1.0001) | 0.14 | 1.00E+00 |
| ALB | 1.0002 (1.0001-1.0004) | -0.02 | 1.78E-01 | 1.0002 (1.0001-1.0003) | -0.03 | 2.35E-02 | 0.9996 (0.9993-0.9998) | 0.07 | 7.05E-03 | 1.0001 (1.0001-1.0002) | -0.28 | 1.05E-02 |
| PLA | 1.0003 (0.9984-1.0022) | -0.02 | 1.00E+00 | 1.0002 (0.9991-1.0013) | -0.03 | 1.00E+00 | 1.0002 (0.9994-1.0009) | -0.02 | 1.00E+00 | 1.0000 (0.9997-1.0004) | -0.07 | 1.00E+00 |
| Composite Indicators |  |  |  |  |  |  |  |  |  |  |  |  |
| AGR | 1.0004 (0.9994-1.0014) | -0.03 | 1.00E+00 | 1.0002 (0.9997-1.0006) | -0.02 | 1.00E+00 | 1.0001 (0.9999-1.0002) | -0.01 | 1.00E+00 | 1.0001 (0.9999-1.0003) | -0.11 | 1.00E+00 |
| AISI | 1.0027 (1.0011-1.0044) | -0.23 | 2.63E-02 | 1.0014 (1.0005-1.0022) | -0.25 | 2.73E-02 | 1.0009 (1.0004-1.0014) | -0.14 | 2.09E-02 | 1.0005 (1.0002-1.0007) | -0.01 | 3.32E-02 |
| CAR | 1.0012 (1.0000-1.0025) | -0.10 | 1.00E+00 | 1.0006 (1.0000-1.0012) | -0.10 | 1.00E+00 | 1.0003 (1.0000-1.0006) | -0.04 | 9.65E-01 | 1.0002 (1.0000-1.0005) | -0.43 | 1.00E+00 |
| dNLR | 1.0005 (1.0001-1.0010) | -0.04 | 5.09E-01 | 1.0003 (1.0000-1.0006) | -0.05 | 4.95E-01 | 1.0002 (1.0000-1.0003) | -0.02 | 4.69E-01 | 1.0001 (1.0000-1.0002) | -0.15 | 5.73E-01 |
| HALP | 0.9983 (0.9967-1.0000) | 0.14 | 9.24E-01 | 0.9992 (0.9983-1.0000) | 0.15 | 1.00E+00 | 0.9996 (0.9992-1.0000) | 0.06 | 1.00E+00 | 0.9996 (0.9992-1.0000) | 0.01 | 6.97E-01 |
| MLR | 0.9990 (0.9982-0.9998) | 0.07 | 3.92E-01 | 0.9994 (0.9989-0.9999) | 0.09 | 4.38E-01 | 0.9997 (0.9995-1.0000) | 0.04 | 5.18E-01 | 0.9998 (0.9996-1.0000) | 0.38 | 3.63E-01 |
| NLPR | 0.9984 (0.9976-0.9992) | 0.12 | 2.02E-03 | 0.9990 (0.9985-0.9995) | 0.15 | 2.24E-03 | 0.9994 (0.9991-0.9997) | 0.08 | 3.43E-03 | 0.9996 (0.9994-0.9998) | 0.51 | 1.64E-03 |
| NLR | 0.9994 (0.9991-0.9997) | 0.05 | 7.22E-03 | 0.9996 (0.9994-0.9998) | 0.07 | 4.72E-03 | 0.9998 (0.9998-0.9999) | 0.02 | 9.07E-03 | 0.9998 (0.9997-0.9999) | 0.55 | 4.08E-03 |
| PLR | 0.9995 (0.9982-1.0009) | 0.04 | 1.00E+00 | 0.9998 (0.9991-1.0005) | 0.03 | 1.00E+00 | 0.9999 (0.9997-1.0002) | 0.01 | 1.00E+00 | 0.9999 (0.9995-1.0002) | 0.21 | 1.00E+00 |
| PNI | 0.9975 (0.9953-0.9997) | 0.19 | 4.92E-01 | 0.9987 (0.9976-0.9999) | 0.22 | 6.10E-01 | 0.9992 (0.9985-1.0000) | 0.11 | 1.00E+00 | 0.9995 (0.9991-0.9999) | 0.91 | 3.79E-01 |
| SII | 1.0012 (1.0005-1.0020) | -0.11 | 3.71E-02 | 1.0007 (1.0002-1.0011) | -0.12 | 3.96E-02 | 1.0005 (1.0002-1.0008) | -0.08 | 3.54E-02 | 1.0002 (1.0001-1.0002) | -0.68 | 5.00E-02 |
| SIRI | 1.0017 (1.0009-1.0026) | -0.14 | 1.48E-03 | 1.0007 (1.0004-1.0011) | -0.13 | 1.65E-03 | 1.0005 (1.0002-1.0007) | -0.07 | 1.40E-03 | 1.0003 (1.0001-1.0004) | -0.57 | 2.39E-03 |
| Indirect effect | LBM | | | WC | | | WHR | | | WHtR | | |
|  | HR (95%CI) | proportion | Adjust *P* | HR (95%CI) | proportion | Adjust *P* | HR (95%CI) | proportion | Adjust *P* | HR (95%CI) | proportion | Adjust *P* |
| Individual indicators | |  |  |  |  |  |  |  |  |  |  |  |
| CRP | 1.0002 (0.9998-1.0006) | 0.04 | 1.00E+00 | 1.0003 (0.9998-1.0009) | -0.26 | 1.00E+00 | 1.0382 (0.9812-1.0984) | -0.18 | 1.00E+00 | 1.0660 (0.9771-1.1631) | -0.11 | 1.00E+00 |
| WBC | 1.0005 (1.0002-1.0009) | 0.09 | 6.60E-02 | 1.0011 (1.0004-1.0017) | -0.94 | 2.95E-02 | 1.1419 (1.0521-1.2393) | -0.79 | 3.15E-02 | 1.2291 (1.0906-1.3852) | -0.38 | 1.51E-02 |
| MO | 1.0002 (0.9999-1.0004) | 0.03 | 1.00E+00 | 1.0003 (0.9999-1.0008) | -0.23 | 1.00E+00 | 1.0414 (0.9886-1.0971) | -0.19 | 1.00E+00 | 1.0660 (0.9894-1.1485) | -0.11 | 1.00E+00 |
| LY | 0.9998 (0.9993-1.0003) | -0.03 | 1.00E+00 | 0.9998 (0.9992-1.0004) | 0.12 | 1.00E+00 | 0.9796 (0.9138-1.0502) | 0.09 | 1.00E+00 | 0.9789 (0.8777-1.0917) | 0.03 | 1.00E+00 |
| NE | 1.0004 (1.0002-1.0006) | 0.07 | 5.71E-03 | 1.0010 (1.0005-1.0015) | -0.89 | 2.74E-03 | 1.1355 (1.0639-1.2120) | -0.76 | 2.76E-03 | 1.2050 (1.0988-1.3214) | -0.35 | 1.56E-03 |
| HEMO | 0.9991 (0.9988-0.9994) | -0.14 | 1.05E-08 | 0.9987 (0.9983-0.9991) | 0.01 | 4.64E-08 | 0.8691 (0.8298-0.9102) | 0.81 | 5.57E-08 | 0.8249 (0.7728-0.8805) | 0.36 | 1.54E-07 |
| GLO | 1.0000 (0.9999-1.0000) | 0.00 | 1.00E+00 | 0.9999 (0.9997-1.0001) | 0.07 | 1.00E+00 | 0.9890 (0.9639-1.0147) | 0.05 | 1.00E+00 | 0.9863 (0.9508-1.0231) | 0.02 | 1.00E+00 |
| ALB | 1.0003 (1.0002-1.0005) | 0.06 | 2.78E-03 | 1.0000 (1.0000-1.0000) | 0.01 | 1.00E+00 | 0.9856 (0.9777-0.9935) | 0.05 | 8.44E-03 | 1.0029 (0.9987-1.0071) | 0.00 | 1.00E+00 |
| PLA | 1.0000 (0.9999-1.0001) | 0.00 | 1.00E+00 | 1.0001 (0.9997-1.0004) | -0.04 | 1.00E+00 | 1.0086 (0.9596-1.0601) | -0.04 | 1.00E+00 | 1.0154 (0.9452-1.0908) | -0.03 | 1.00E+00 |
| Composite Indicators |  |  |  |  |  |  |  |  |  |  |  |  |
| AGR | 1.0000 (0.9999-1.0002) | 0.01 | 1.00E+00 | 1.0001 (0.9999-1.0003) | -0.05 | 1.00E+00 | 1.0077 (0.9884-1.0275) | -0.03 | 1.00E+00 | 1.0146 (0.9825-1.0478) | -0.02 | 1.00E+00 |
| AISI | 1.0001 (1.0000-1.0001) | 0.01 | 1.36E-01 | 1.0005 (1.0002-1.0009) | -0.44 | 2.71E-02 | 1.0826 (1.0319-1.1358) | -0.39 | 2.46E-02 | 1.1145 (1.0456-1.1879) | -0.19 | 1.84E-02 |
| CAR | 1.0002 (1.0000-1.0003) | 0.03 | 1.00E+00 | 1.0002 (1.0000-1.0005) | -0.17 | 1.00E+00 | 1.0274 (0.9996-1.0559) | -0.13 | 1.00E+00 | 1.0420 (1.0006-1.0850) | -0.07 | 9.76E-01 |
| dNLR | 1.0000 (0.9999-1.0000) | 0.00 | 1.00E+00 | 1.0001 (1.0000-1.0002) | -0.08 | 4.97E-01 | 1.0173 (1.0023-1.0325) | -0.09 | 4.87E-01 | 1.0247 (1.0034-1.0464) | -0.04 | 4.77E-01 |
| HALP | 0.9996 (0.9992-0.9999) | -0.07 | 3.79E-01 | 0.9997 (0.9994-1.0000) | 0.24 | 7.82E-01 | 0.9679 (0.9390-0.9976) | 0.14 | 7.21E-01 | 0.9505 (0.9042-0.9992) | 0.08 | 9.79E-01 |
| MLR | 0.9998 (0.9996-1.0000) | -0.04 | 2.81E-01 | 0.9998 (0.9997-1.0000) | 0.11 | 3.67E-01 | 0.9864 (0.9754-0.9976) | 0.06 | 3.74E-01 | 0.9703 (0.9463-0.9949) | 0.05 | 3.81E-01 |
| NLPR | 0.9998 (0.9996-0.9999) | -0.04 | 1.48E-03 | 0.9997 (0.9996-0.9999) | 0.19 | 1.80E-03 | 0.9738 (0.9608-0.9869) | 0.12 | 2.09E-03 | 0.9518 (0.9283-0.9760) | 0.08 | 2.35E-03 |
| NLR | 0.9997 (0.9995-0.9998) | -0.06 | 2.09E-03 | 0.9999 (0.9999-1.0000) | 0.06 | 2.09E-02 | 1.0062 (1.0015-1.0110) | -0.03 | 1.98E-01 | 0.9935 (0.9885-0.9986) | 0.01 | 2.54E-01 |
| PLR | 0.9998 (0.9995-1.0002) | -0.03 | 1.00E+00 | 0.9999 (0.9996-1.0002) | 0.07 | 1.00E+00 | 0.9909 (0.9673-1.0150) | 0.04 | 1.00E+00 | 0.9866 (0.9462-1.0287) | 0.02 | 1.00E+00 |
| PNI | 0.9997 (0.9995-0.9999) | -0.05 | 2.64E-01 | 0.9995 (0.9991-0.9999) | 0.32 | 4.18E-01 | 0.9385 (0.8901-0.9894) | 0.24 | 3.90E-01 | 0.9185 (0.8526-0.9894) | 0.13 | 5.26E-01 |
| SII | 0.9998 (0.9997-0.9999) | -0.03 | 3.84E-02 | 1.0003 (1.0001-1.0004) | -0.21 | 3.46E-02 | 1.0477 (1.0181-1.0782) | -0.23 | 3.01E-02 | 1.0589 (1.0224-1.0966) | -0.10 | 2.89E-02 |
| SIRI | 1.0000 (1.0000-1.0000) | 0.00 | 1.00E+00 | 1.0004 (1.0002-1.0006) | -0.28 | 1.43E-03 | 1.0618 (1.0311-1.0934) | -0.28 | 1.29E-03 | 1.0753 (1.0383-1.1137) | -0.12 | 1.04E-03 |

Indirect effect represents indirect effect of mediation analysis of systemic inflammatory markers in the associations between anthropometric measurements and obesity-related cancer incidence.

457,806 participants in the MJ Cohort study (1996-2008)

Participants under the age of 18 years old (n=14,797)

Participants with prevalent cancer* (n=6,040)

Participants with less than one year follow-up (n=1,238)

435,731 participants

Participants with missing data in C-reactive Protein (CRP), albumin, globulin, platelets and white blood cells counts (n=79,177)

356,554 participants included in the main analysis

**Figure S1**. Flowchart of the study population inclusion

* Participants diagnosed with cancer prior to the recruitment date into MJ cohort or had self-reported cancer records.


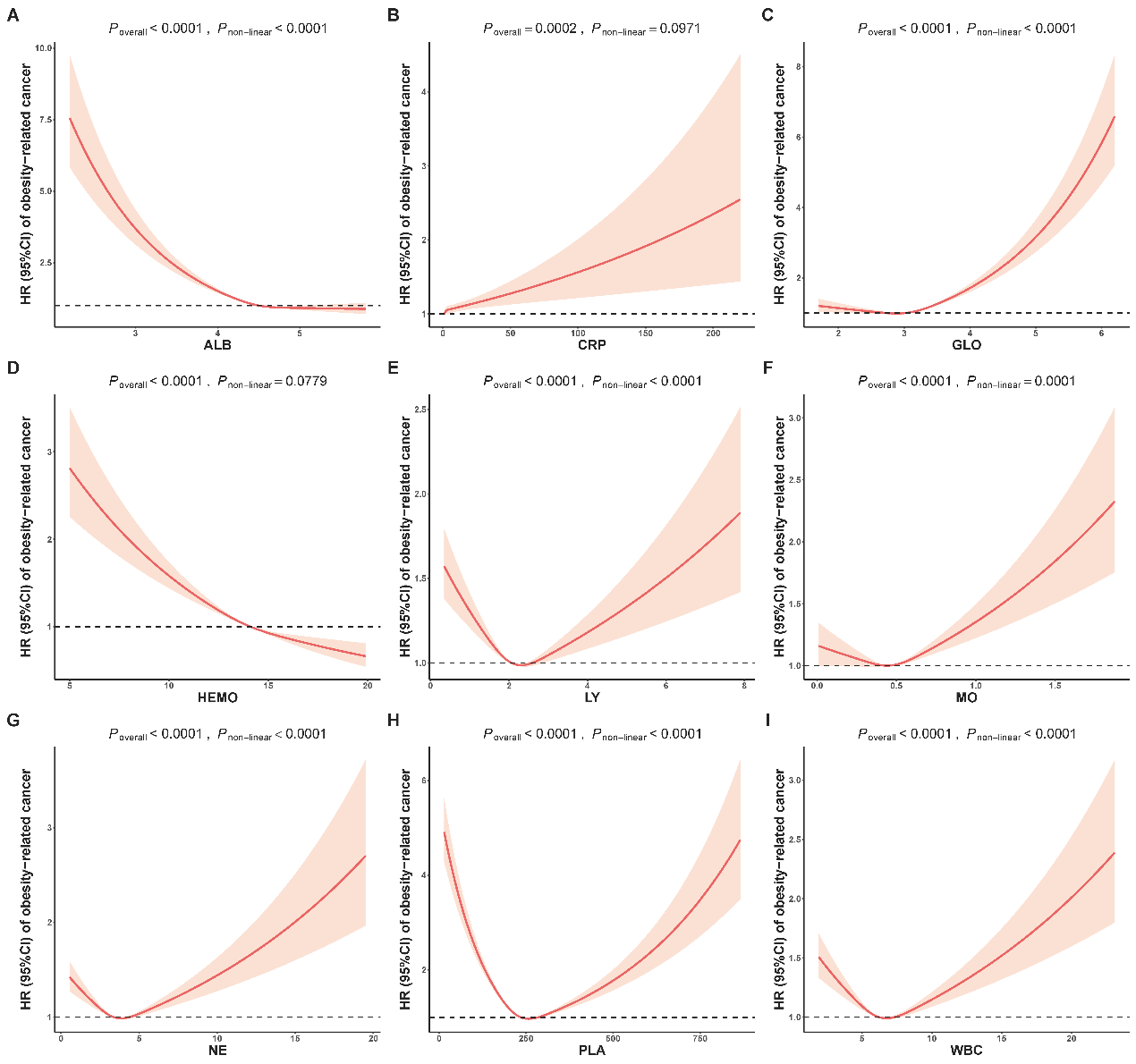


# Figure S2. Analysis of the shape of the relationship between individual inflammatory biomarkers and obesity related cancer incidence using restricted cubic spline.

ALB, albumin; CRP, C-reactive Protein; GLO, globulin; HEMO, hemoglobin; LY, lymphocyte; MO, monocyte; NE, neutrophil; PLA, platelets; WBC, white blood cells; Hazard ratios, HR.


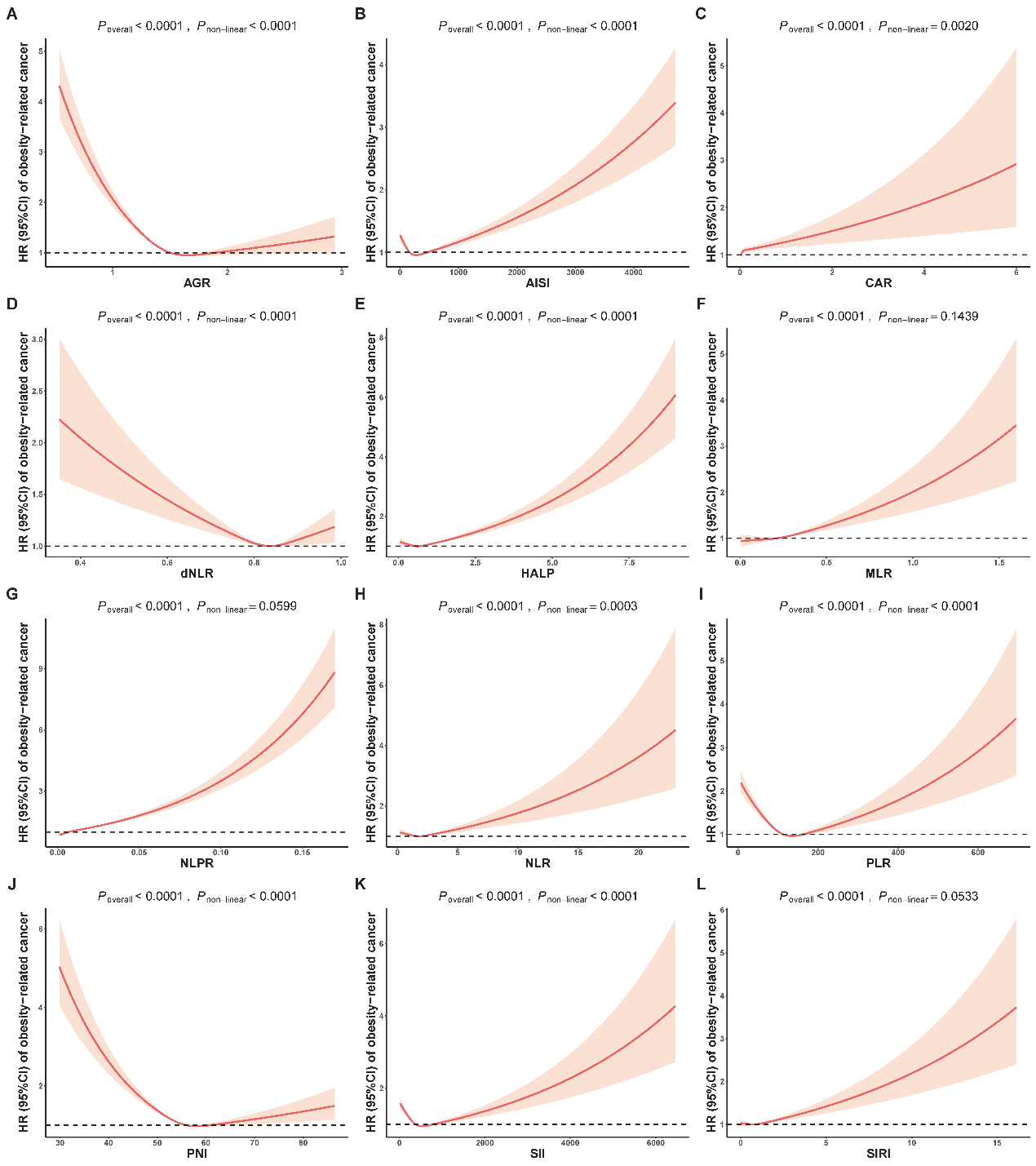


**Figure S3. Analysis of the shape of the relationship between composite inflammatory biomarkers and obesity related cancer incidence using restricted cubic spline.** AGR, Albumin/Globulin ratio; AISI, aggregate index of systemic inflammation; CAR, C-reactive Protein/Albumin; dNLR, derived NLR; HALP, The hemoglobin, albumin, lymphocyte, and platelet score; MLR, monocyte-lymphocyte ratio; NLPR, neutrophil to lymphocyte × platelet ratio; NLR, neutrophil-to-lymphocyte ratio; PLR, platelet-to-lymphocyte ratio; PNI, Prognostic Nutritional Index; SII, systemic immune-inflammation index; SIRI, systemic inflammation response index; Hazard ratios, HR.


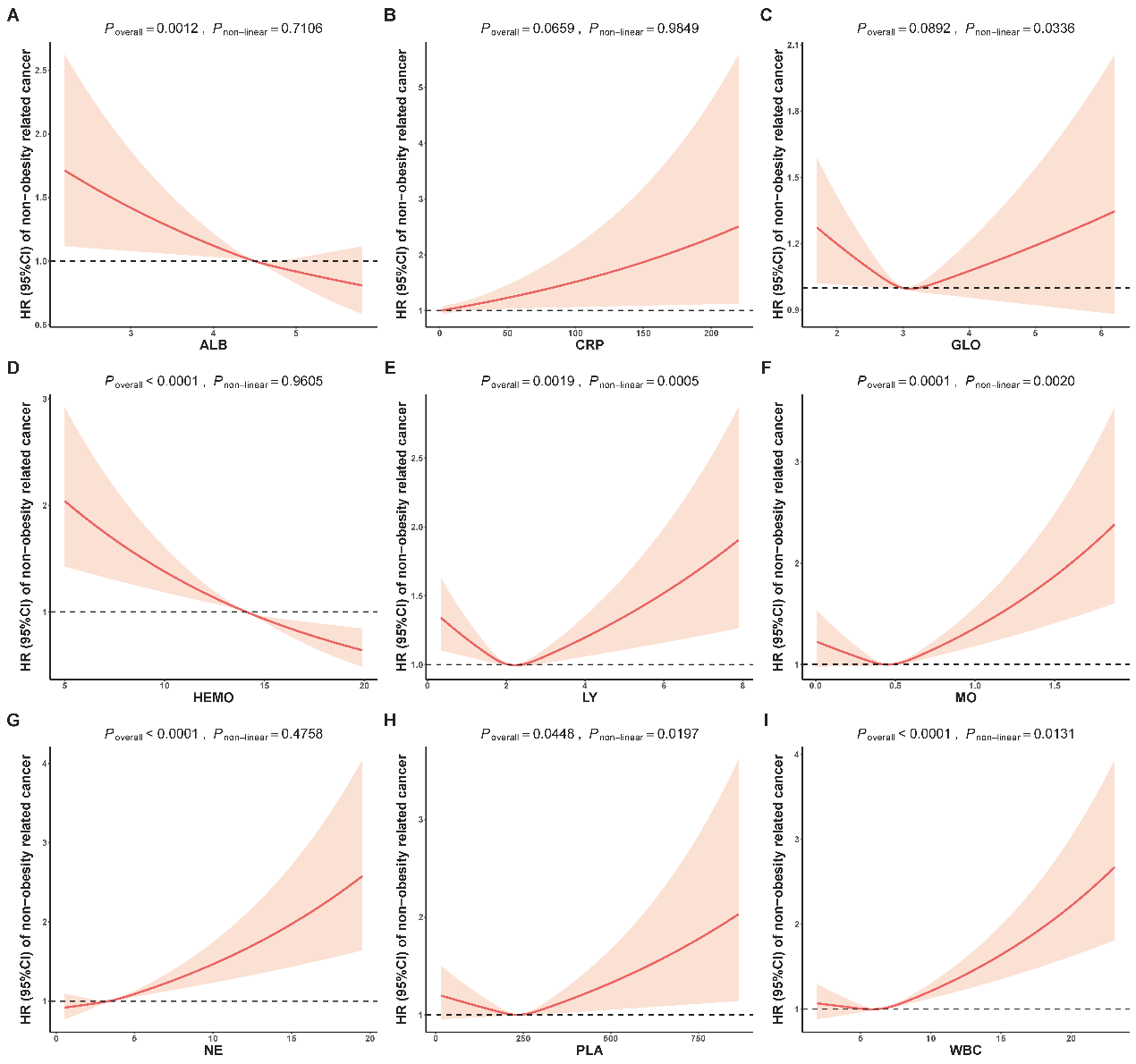


# Figure S4. Analysis of the shape of the relationship between individual inflammatory biomarkers and non-obesity related cancer incidence using restricted cubic spline.

ALB, albumin; CRP, C-reactive Protein; GLO, globulin; HEMO, hemoglobin; LY, lymphocyte; MO, monocyte; NE, neutrophil; PLA, platelets; WBC, white blood cells; Hazard ratios, HR.


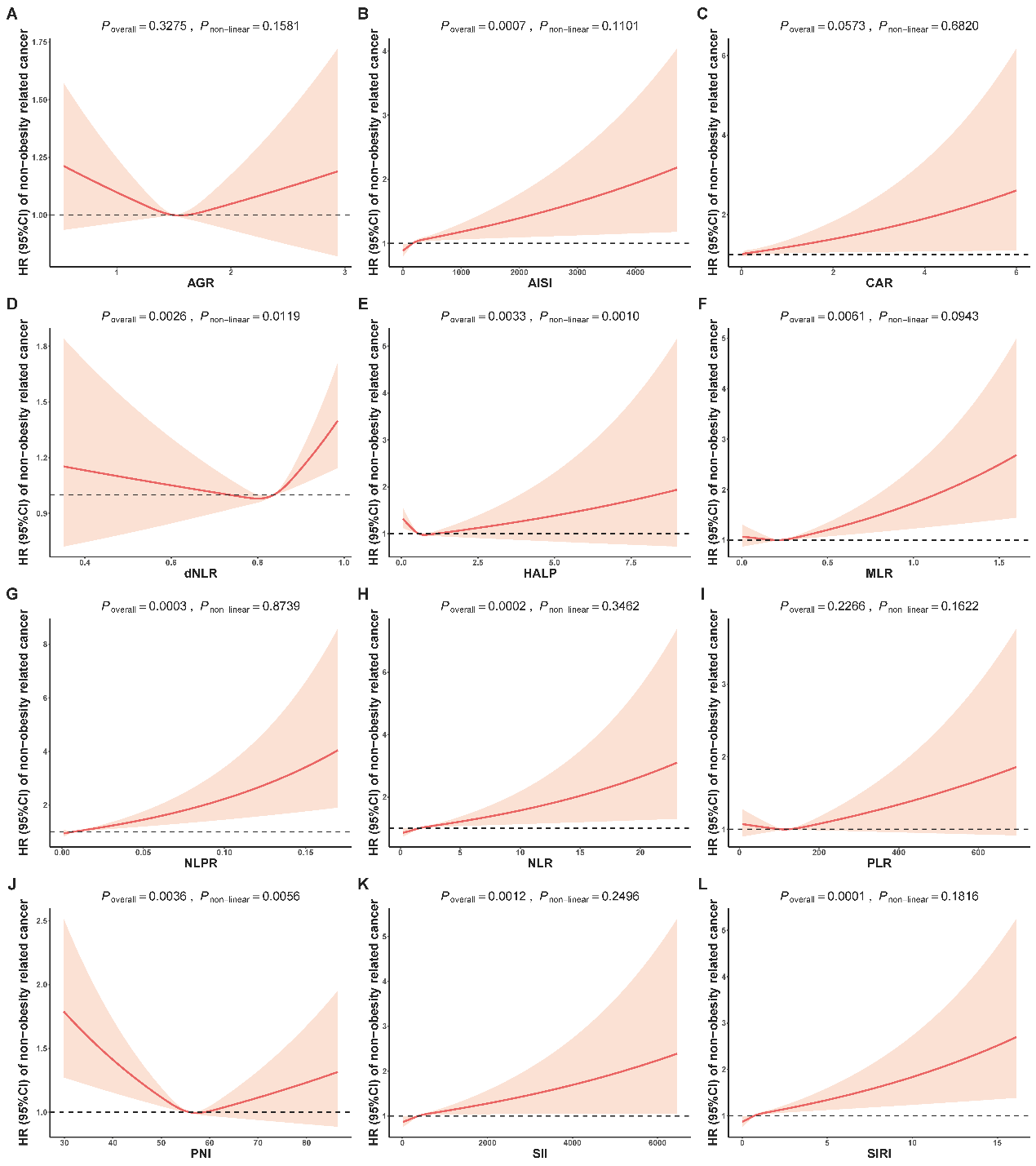


**Figure S5. Analysis of the shape of the relationship between composite inflammatory biomarkers and non-obesity related cancer incidence using restricted cubic spline.** AGR, Albumin/Globulin ratio; AISI, aggregate index of systemic inflammation; CAR, C-reactive Protein/Albumin; dNLR, derived NLR; HALP, The hemoglobin, albumin, lymphocyte, and platelet score; MLR, monocyte-lymphocyte ratio; NLPR, neutrophil to lymphocyte × platelet ratio; NLR, neutrophil-to-lymphocyte ratio; PLR, platelet-to-lymphocyte ratio; PNI, Prognostic Nutritional Index; SII, systemic immune-inflammation index; SIRI, systemic inflammation response index; Hazard ratios, HR.
